# Identification of molecular targets of *Hypericum* *perforatum* in blood for major depressive disorder: a machine-learning pharmacological study

**DOI:** 10.1186/s13020-024-01018-5

**Published:** 2024-10-09

**Authors:** Zewen Xu, Ayana Meegol Rasteh, Angela Dong, Panpan Wang, Hengrui Liu

**Affiliations:** 1 The First Affiliated Hospital of Jinan University, Guangzhou, China; 2 Cancer Research Institute, Jinan University, Guangzhou, China; 3 Tianjin Yinuo Biomedical Co., Ltd, Tianjin, China; 4 School of Basic Medical Sciences, Guangzhou University of Chinese Medicine, Guangzhou, China; 5 Archbishop Mitty High School, San Jose, CA USA; 6 Havergal College, Toronto, Canada

**Keywords:** *Hypericumperforatum*, Major depressive disorder, Machine learning, Pharmacological targets

## Abstract

**Background:**

Major depressive disorder (MDD) is one of the most common psychiatric disorders worldwide. *Hypericum* *perforatum* (HP) is a traditional herb that has been shown to have antidepressant effects, but its mechanism is unclear. This study aims to identify the molecular targets of HP for the treatment of MDD.

**Methods:**

We performed differential analysis and weighted gene co-expression network analysis (WGCNA) with blood mRNA expression cohort of MDD and healthy control to identify DEGs and significant module genes (gene list 1). Three databases, CTD, DisGeNET, and GeneCards, were used to retrieve MDD-related gene intersections to obtain MDD-predicted targets (gene list 2). The validated targets were retrieved from the TCMSP database (gene list 3). Based on these three gene lists, 13 key pathways were identified. The PPI network was constructed by extracting the intersection of genes and HP-validated targets on all key pathways. Key therapeutic targets were obtained using MCODE and machine learning (LASSO, SVM-RFE). Clinical diagnostic assessments (Nomogram, Correlation, Intergroup expression), and gene set enrichment analysis (GSEA) were performed for the key targets. In addition, immune cell analysis was performed on the blood mRNA expression cohort of MDD to explore the association between the key targets and immune cells. Finally, molecular docking prediction was performed for the targets of HP active ingredients on MDD.

**Results:**

Differential expression analysis and WGCNA module analysis yielded 933 potential targets for MDD. Three disease databases were intersected with 982 MDD-predicted targets. The TCMSP retrieved 275 valid targets for HP. Separate enrichment analysis intersected 13 key pathways. Five key targets (AKT1, MAPK1, MYC, EGF, HSP90AA1) were finally screened based on all enriched genes and HP valid targets. Combined with the signaling pathway and immune cell analysis suggested the effect of peripheral immunity on MDD and the important role of neutrophils in immune inflammation. Finally, the binding of HP active ingredients (quercetin, kaempferol, and luteolin) and all 5 key targets were predicted based on molecular docking.

**Conclusions:**

The active constituents of *Hypericum* *perforatum* can act on MDD and key targets and pathways of this action were identified.

**Supplementary Information:**

The online version contains supplementary material available at 10.1186/s13020-024-01018-5.

## Introduction

Major Depressive Disorder (MDD) is recognized worldwide as a complex and seriously life-altering psychological disorder [1]. Typical clinical symptoms include depressed mood and loss of pleasure or interest in activities, and in severe cases can lead to suicidal behavior. Although many antidepressants are available to alleviate mild and moderate depressive symptoms, there are limitations in major depressive disorder, including low efficacy and high side effects [2]. It is therefore important to find more reliable and targeted treatments for depression [3, 4].

A great deal of current research into depression revolves around the neurotransmitter doctrine, with most antidepressant approaches targeting monoamine neurotransmitters (5-hydroxytryptamine, dopamine, and norepinephrine) for treatment [5, 6]. This has been shown to be the substance that directly affects mood [7], in addition to the other direction of immune inflammation [8]. Inflammation is a manifestation of immune system activation, and there is growing evidence that the development of MDD is associated with immune activation, such as elevated levels of pro-inflammatory cytokines like IL-6 and TNF, which decrease after treatment [9, 10]. It has been shown that traditional antidepressants have anti-inflammatory effects and that the effectiveness of treatment depends on the different immune phenotypes.

Traditional medicine has been applied for therapy for many human diseases [11–19]. *Hypericum* *perforatum* (HP) has been used in herbal medicine and traditional medicine for centuries. Some studies have shown that HP has a superior effect to placebo and is even comparable to standard antidepressants in patients with MDD, with high efficacy and safety in mild and moderate depression and low discontinuation rates [20, 21].

This study aims to identify the molecular targets of HP for the treatment of MDD. In this study, bioinformatics approaches such as differential expression analysis, WGCNA, KEGG pathway analysis, and machine learning were used to explore the mechanism of action and molecular targets of HP for MDD in combination with signaling pathways and immune cells.

## Materials and methods

### The overall design of this study

**Fig. 1:**
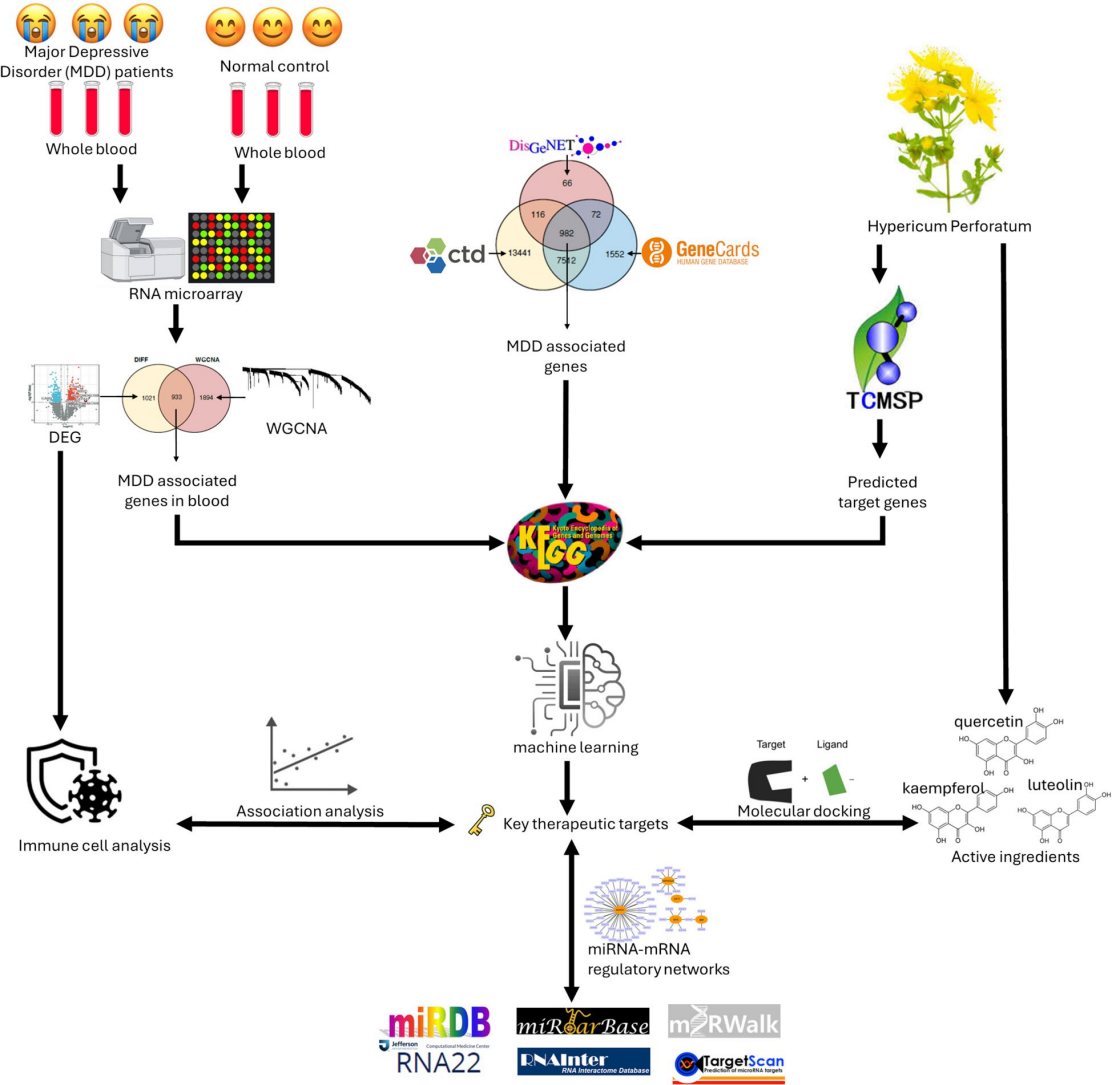
Graphical workflow of this study. **A** Identification of the workflow for the treatment of major depressive disorder with *Hypericum* *perforatum*. **B** Analysis of the databases, software, and tools used

We performed differential analysis and weighted gene co-expression network analysis (WGCNA) with blood mRNA expression cohort of MDD to identify DEGs and significant module genes (gene list 1). Three databases, CTD [22], DisGeNET [23], and GeneCards [24], were used to retrieve MDD-related gene intersections to obtain MDD-predicted targets (gene list 2). The validated targets were retrieved from the TCMSP database [25] (gene list 3). Based on these three gene lists, 13 key pathways were identified. The PPI network was constructed by extracting the intersection of genes and HP-validated targets on all key pathways. Key therapeutic targets were obtained using MCODE [26] and machine learning (LASSO [27], SVM-RFE [28]). Clinical diagnostic assessments (Nomogram, ROC, Correlation, Intergroup expression), and gene set enrichment analysis (GSEA) were performed for the key targets. In addition, immune cell analysis was performed on the blood mRNA expression cohort of MDD to explore the association between the key targets and immune cells. Finally, molecular docking prediction was performed for the targets of HP active ingredients on MDD (Fig. 1).

### Blood cohort description

The cohort of this study involved 128 whole blood samples including 64 MDD patients with generalized anxiety disorder, diagnosed by the MINI questionnaire) and 64 healthy controls. RNA isolation was performed using the standard PAXgene protocol on the Qiagen Biorobot 8000, ensuring good quality RNA for all samples, as confirmed by Agilent Bioanalyzer. The RNA yield ranged from 0.86 to 15.05 ug from each sample, with an average of 6.25 ug. The female-to-male ratio of this cohort is 3:1. 50 ng of RNA from each sample was converted into a biotin-labeled cDNA probe using NuGEN SPIA amplification. These probes were then hybridized to Affymetrix U133_Plus2.0 Genechips. The microarray data were accessed from the GPL570 (Affymetrix Human Genome U133 Plus 2.0 Array) platform and were previously published as GSE98793 [29] in the GEO database (https://www.ncbi.nlm.nih.gov/geo/).

### Identification of differentially expressed genes in patients with major depressive disorder

Differential expression analysis was performed with the limma package of Rstudio (version 4.2.1) and regarding the difference density plot we retained DEGs with logFC > 0.1, p < 0.05, and visualized volcano plots for logFC > 0.1, adj. P < 0.01 [30].

### Screening of potential target genes by weighted gene co-expression network analysis

The first 6000 differentially expressed genes from the MDD and healthy control groups of blood microarray data were taken to construct co-expression networks using the WGCNA package [31]. This was then converted into a topological overlap matrix (TOM) using hierarchical clustering to identify panels and calculate signature genes. Correlations between each module and MDD samples or normal samples were assessed to select key modules as MDD-associated module genes. The key module genes were intersected with the Venn package of DEGs in Rstudio(version 4.2.1) to obtain potential target genes for MDD.

### Screening for predictive target genes in *Hypericum perforatum* and predictive target genes in major depression

The active ingredients were selected according to the Traditional Chinese Medicine Systematic Pharmacology (TCMSP) [25] database (https://old.tcmsp-e.com/tcmsp.php) to meet oral bioavailability (OB) ≥ 30% and drug similarity (DL) ≥ 0.18, and their active ingredient prediction targets were collected [25]. Relevant targets for MDD were collected in the CTD database [22] (http://ctdbase.org/), GeneCards database (https://www.genecards.org/), and DisGeNET database [23] (https://www.disgenet.org/) and the Venn package intersection in Rstudio (version 4.2.1) was used as a predictive target for MDD.

### Target gene pathway enrichment analysis and screening of predicted therapeutic target genes

Microarray data differential expression analysis and WGCNA analysis based on the clusterProfiler package for Rtudio (version 4.2.1) were performed with multiple comparison corrections (adjP < 0.05 as cut-off value) and intersected to obtain MDD potential target genes [32]. Target genes for HP were predicted in the TCMSP database. Three disease databases were intersected for MDD-predicted target genes. KEGGa, KEGGb, and KEGGc were obtained by enrichment pathway analysis. venn package based on Rstudio (version 4.2.1) was used to intersect the results of these three pathways to obtain key pathways and calculate enrichment numbers. All genes on the pathways were collected in the WEB-based Kyoto Encyclopedia of Genes and Genomes (KEGG) database (https://www.genome.jp/kegg/) and intersected with the HP predicted target genes to obtain the predicted therapeutic target genes. In addition, we visualized key pathways based on the preview package [33].

### Protein–protein interaction networks and constructing component-pathway-target networks

To further explore the interactions between predicted therapeutic target genes, PPI networks were constructed using the STRING database [34], and the lowest interaction score above 0.4 was considered significant. The PPI visualization network was then done using Cytoscape software [35]. Key protein expression sub-networks were screened using the Cytoscape plugin Minimum Common Oncology Data Element (MCODE) [26]. In addition, we constructed networks and visualized them in Cytoscape software for HP active ingredients, HP-predicted target genes, and key pathways for target enrichment.

### Screening key targets for MDD patients by machine learning algorithms

Two machine learning algorithms were used in this study. The glmnet package of Rstudio (version 4.2.1) was used to perform Lasso regression analysis on the genes of the key subnetwork with a random seed of “123456” [36]. SVM-RFE analysis was performed on the genes of the key subnetworks using the e1071 package of Rstudio (version 4.2.1) with a random seed of “1234567890”. A 10× cross-validation was applied to validate the model. The genes obtained from the intersection of the two analyses were considered potential therapeutic markers for MDD patients. In addition, a column line graph based on potential therapeutic markers was constructed using the rms package, receiver operating characteristic (ROC) analysis was performed on blood RNA microarray data, and the AUC values of these five pivotal genes were calculated using the pROC package to assess their clinical diagnostic value and visualized [37].

### Characterization of key targets for expression, correlation, and gene set enrichment analysis

The expression of key targets was correlated and visualized based on the corrplot package in Rstudio (version 4.2.1). The expression levels of each pivotal gene were analyzed based on the Wilcoxon rank sum test. GSEA analysis was then performed on each pivotal gene to further understand the function of the enriched pathway.

### Immune cell analysis

It has been shown that the pathophysiology of MDD is closely related to the immune system. Especially, our data are from blood that might include strong blood cell signals. Therefore, immune cell analysis of blood RNA microarray data using the CIBERSORT package based on 22 different immune cells was performed to correlate potential therapeutic markers with 22 immune cells [38]. CIBERSORT is a robust computational approach designed to measure the proportions of cells in bulk samples using gene expression profiles (GEPs). By integrating support vector regression and leveraging pre-existing data on expression profiles from distinct leukocyte subsets, CIBERSORT effectively determines the immune cell composition within a bulk sample. The expression of potential therapeutic markers in 22 immune cells was also analyzed using WEB-based CIBERSORTx (https://cibersortx.stanford.edu/) to further understand the effect of MDD on specific immune cells.

### Construction of key target miRNA-mRNA regulatory networks

In order to further explore the mechanism of action of key targets in depression under the condition of minimizing the false positive prediction rate. miRNA predictions for key targets were performed based on six databases: miRDB [39], miRTarBase [39], miRWalk [40], RNA22 [41], RNAInter [42], and TargetScan [43]. The upset package of Rstudio (version 4.2.1) was used to take intersections and construct miRNA-mRNA networks based on Cytoscape software.

### Molecular docking verification

Combined PDB and UniProt databases to obtain crystal structures of key targets, pre-processed using AutoDockTools (version 1.5.6) [36, 44]. The PDB files used in this analysis were 2OJG [45], 1JL9 [46], 1UY6 [47], 1H10 [48], and 5I4Z [49]. The detailed information for these protein structures are provided in S-Table 1. The 3D structures of the small molecules of the drug Throughgut were retrieved and downloaded in TCMSP. We then ran AutoDock for molecular docking of macromolecules and ligands according to default parameters. Each pair of docking was conducted nine times and the lowest binding affinity was recorded. The docking models were visualized and the binding energy was displayed in a heat map using the pheatmap package in Rstudio (version 4.2.1).

## Results

### Identification of differentially expressed genes and WGCNA construction in MDD patient blood

**Fig. 2:**
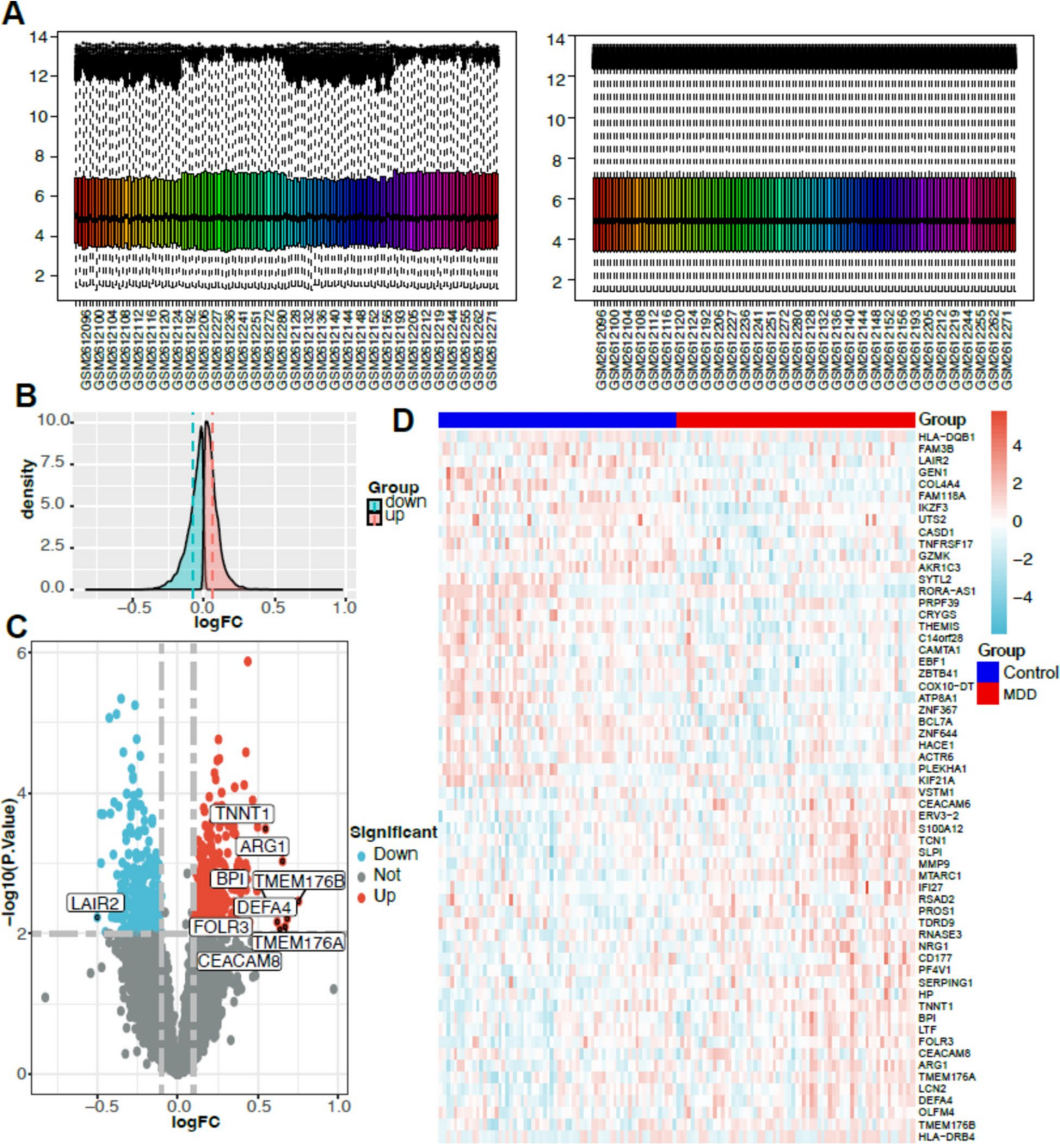
Identification of DEG in peripheral blood of patients with MDD. **A** Normalization process. **B** Sample expression density plot, pink indicates depression up-regulated gene expression and cyan indicates depression down-regulated gene expression. Where the dashed lines represent the mean of each of the two groups. **C** Volcano plot showing differential genes in MDD, red is for significantly up-regulated genes and blue is for significantly down-regulated genes. **D** Heat map showing the expression of the top 30 differential genes in the sample

The blood RNA microarray data were standardized and analyzed for differences (Fig. 2A), and a final screen (logFC > 0.01, P < 0.05) was performed to obtain 1954 differential genes, of which 909 were down-regulated and 1045 were down-regulated (Fig. 2C, D), according to the difference density distribution plot (Fig. 2B).

**Fig. 3:**
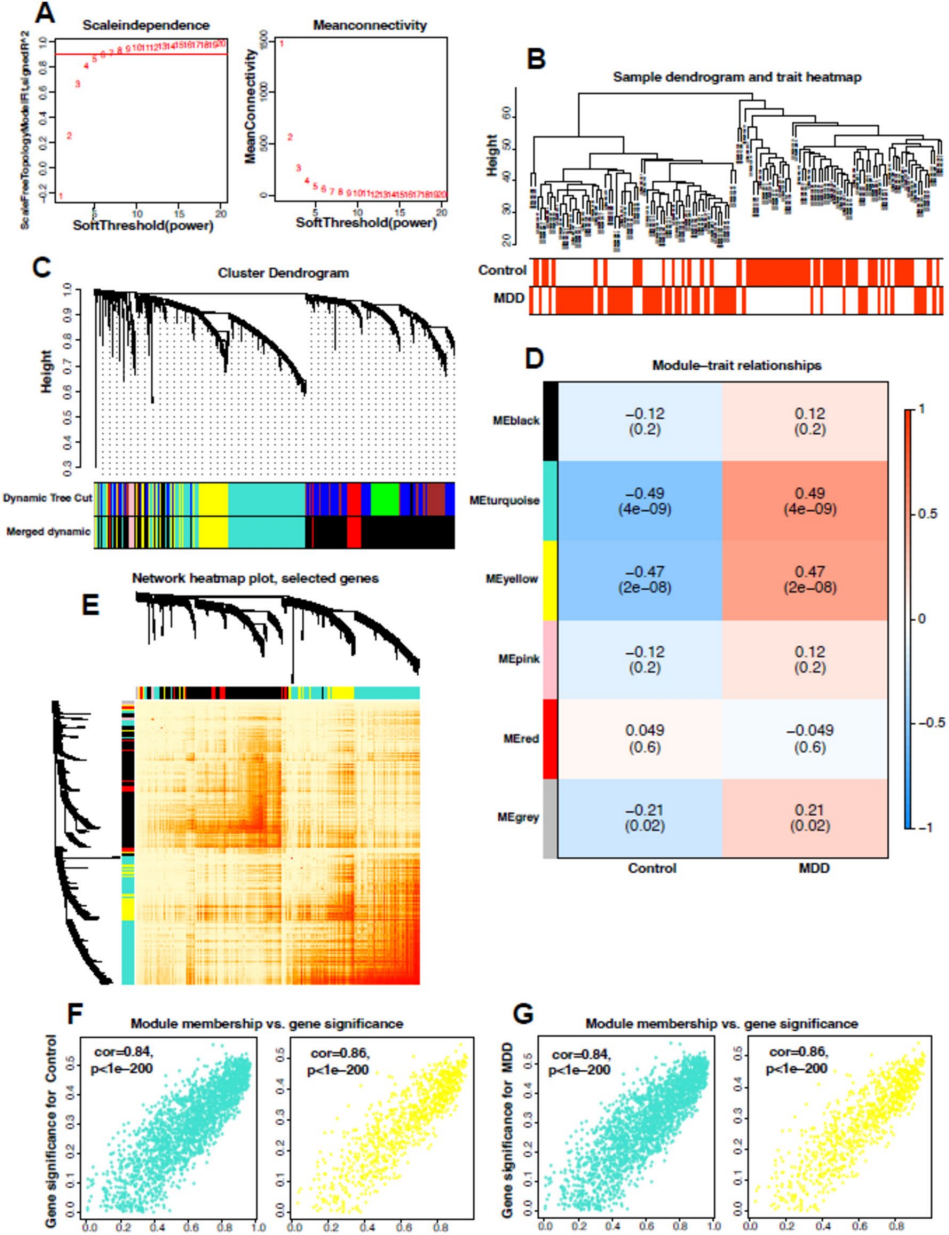
Construction of the weighted gene co-expression network for MDD. **A** Soft threshold β = 5 and scale-free topological fit index (R2). **B** Sample clustering dendrograms with leaves corresponding to each sample. **C** Original and combined modules at a dynamic cut height of 0.2. **D** Heat map of module-trait correlations. 5 rows correspond to each of the 5 combined modules, 2 columns correspond to the normal and MDD groups, and the cells contain the corresponding correlation coefficients and P values. **E** Cluster dendrogram of the module trait genes. **F** MM-GS scatter plots of peacock green and yellow modules in the control group. **G** Scatterplot of peacock green module and blue module MM-GS of MDD group

**Fig. 4:**
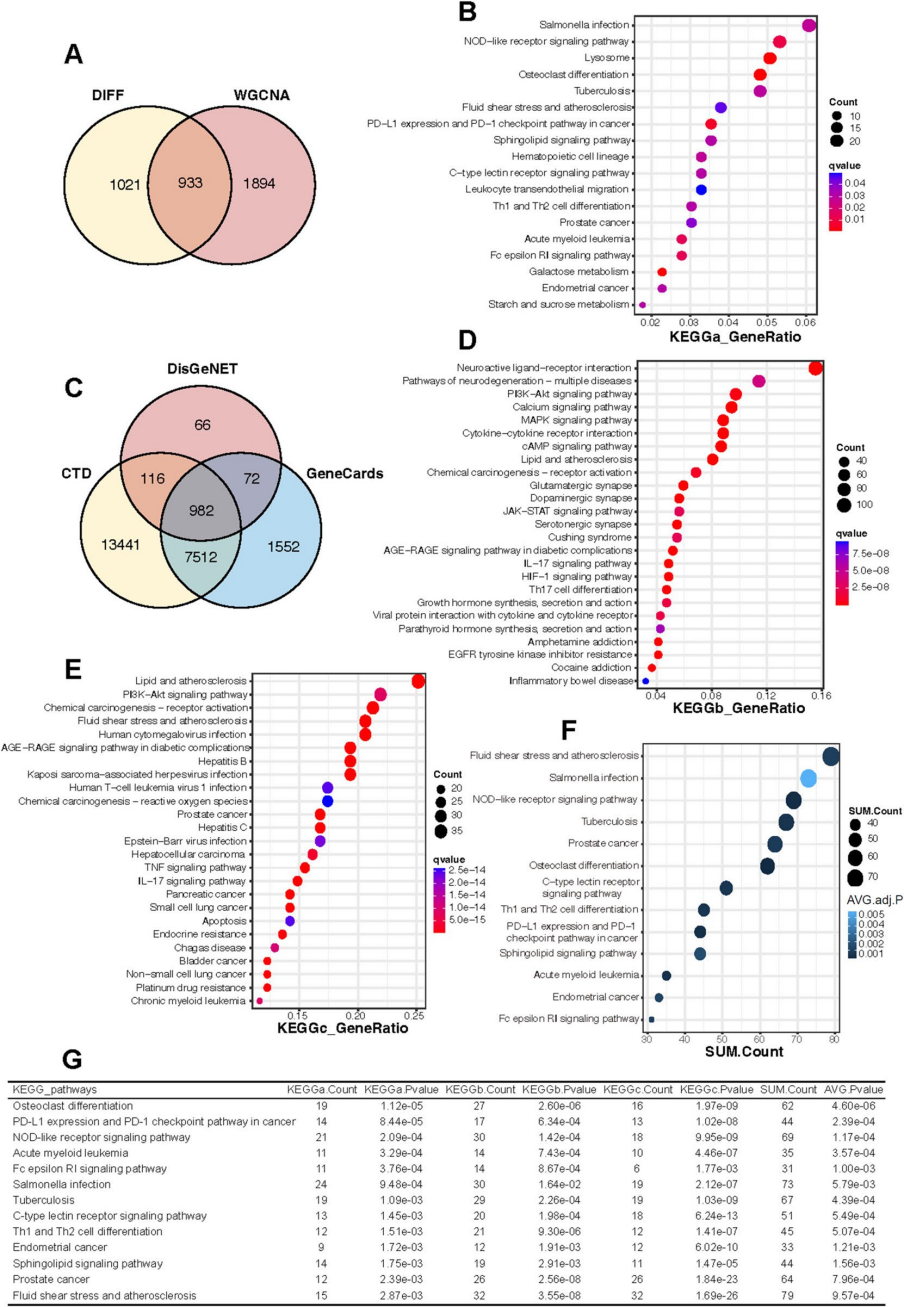
Identification of key KEGG pathways. **A** DEGs mapped to WGCNA key module genes. **B** KEGG enrichment results for KEGGa of intersecting genes of DEGs and WGCNA key module genes. **C** CTD, DisGeNET, GeneCards database predicted gene intersection results. **D** KEGG enrichment results for KEGGb of cross-linked genes in the disease database. **E** KEGG enrichment results for KEGGc of active ingredient targets in *Hypericum* *perforatum*. **F** KEGGa, KEGGb and KEGGc enrichment results for cross-linked key pathways. **G** Number and P-value of enriched genes for the 13 key KEGG pathways

The expression values of the top 6000 differential genes were selected to construct a co-expression network using WGCNA. The samples were clustered according to Pearson's correlation coefficient to obtain a sample clustering tree, and an optimal soft threshold of 5 (R-based scale-free topology criterion^2^ = 0.9) was chosen to obtain a scale-free network (Fig. 3A, B). A total of five modules (2731 black, 20 grey, 117 pink, 305 red, 2005 turquoise, 822 yellow) were obtained by merging dynamic modules with DissThres set to 0.2 (Fig. 3C). MM and GS between modules and MDD were calculated and correlation heat maps were drawn (Fig. 3D–G). We selected genes from the peacock blue module and the yellow module to intersect with DEGs and obtained 933 potential targets associated with MDD (Fig. 4A).

### Identification of the key pathways in HP based on three KEGG enrichments

A total of 275 active ingredient targets for HP (with OB > 30 and DL > 0.18) were obtained from the TCMSP database. The predicted target genes related to depression were crossed in three databases CTD, DisGeNET, and GeneCards respectively using MDD as the keyword, and 982 predicted targets for MDD were obtained (Fig. 4C). We then performed KEGG pathway enrichment analysis on (1) the crossover genes of differential genes and WGCNA key module genes, (2) predicted MDD targets from the three databases, and (3) HP active ingredient targets respectively to obtain KEGGa, KEGGb, and KEGGc (Fig. 4B, D, E). Finally, we crossed these three enriched pathways to obtain 13 key pathways and visualized the bubble plots (Fig. 4F, G).

### Constructing component-target-pathway networks and PPI networks and selecting key networks based on MCODE

**Fig. 5:**
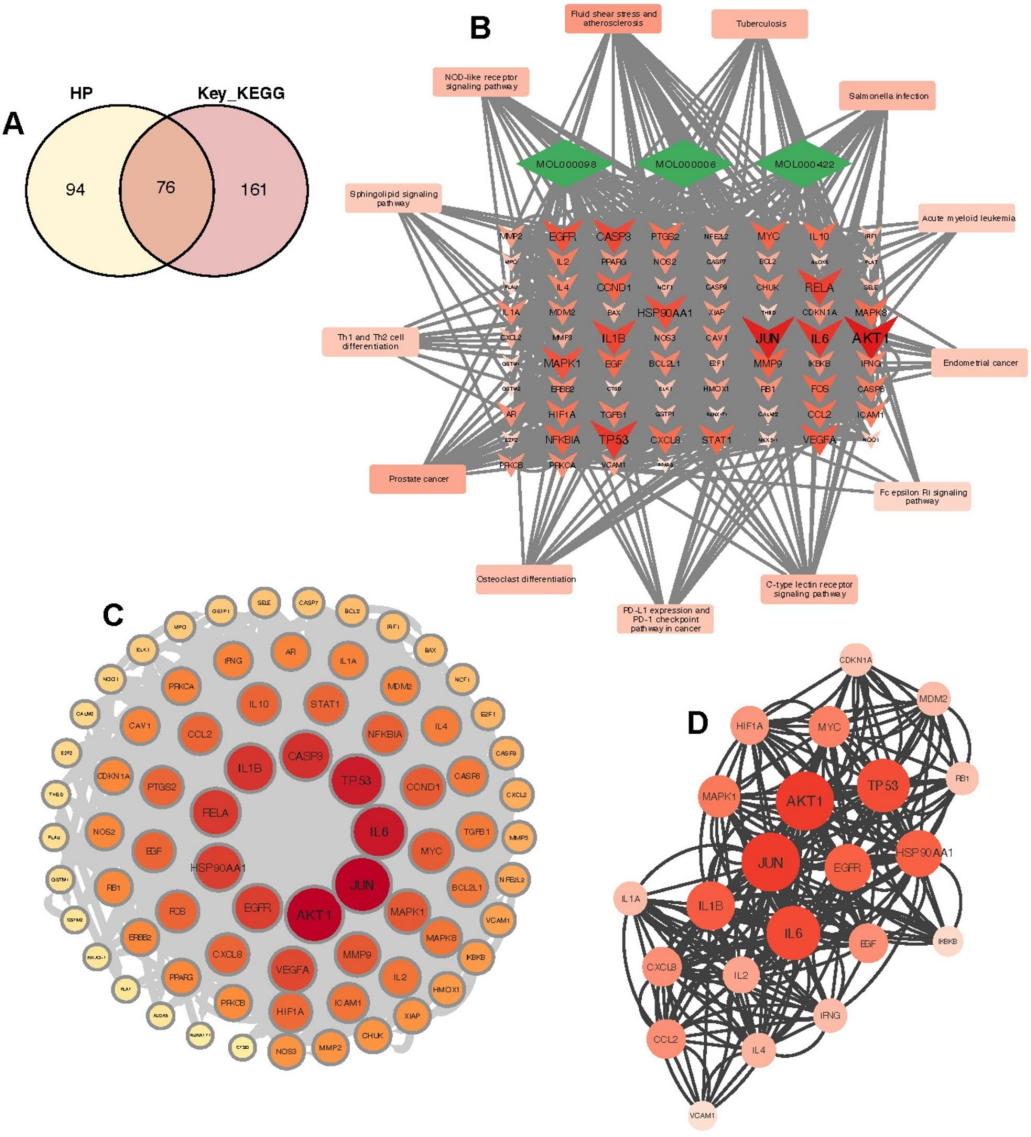
Network construction for predicting therapeutic target genes. **A** Results of the intersection of all genes in the active ingredient target and key KEGG pathway of *Hypericum* *perforatum*. **B** The “component-target-pathway” network, with the rectangular node being the key KEGG pathway, the diamond node being the active ingredient of *Hypericum* *perforatum*, and the triangular node being the 76 predicted therapeutic target genes, with the darker colour and larger shape indicating higher association. **C** PPI network constructed from 76 predicted therapeutic target genes. **D** The key network obtained based on MCODE

All genes enriched in the 13 key pathways were combined and merged with the active ingredient targets of HP to obtain 76 predicted therapeutic target genes (Fig. 5A). A “component-target-pathway” network was then constructed (Fig. 5B). A protein interaction network was constructed, consisting of 76 nodes and 1426 lines (Fig. 5C). The key network was finally screened using the MCODE plugin and contained 22 candidate key genes (IL2, CXCL8, MAPK1, JUN, AKT1, IL4, IL1A, VCAM1, MYC, EGFR, IFNG, TP53, HIF1A, RB1, IKBKB, EGF, IL6, IL1B, CCL2, CDKN1A. HSP90AA1, MDM2) (Fig. 5D).

### Machine learning-based algorithm screens for 5 key targets for MDD patients

**Fig. 6:**
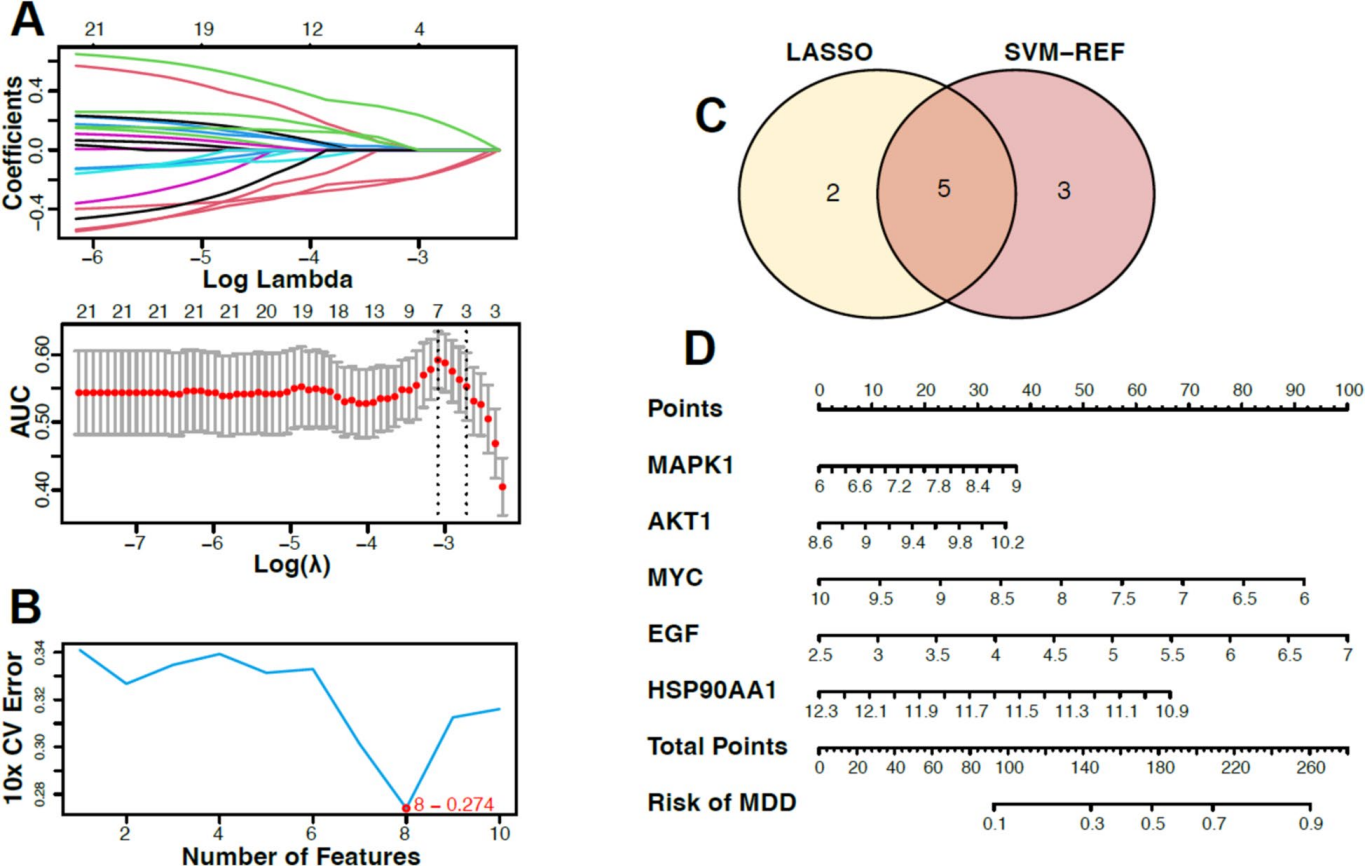
Machine learning-based screening of key targets. **A** LASSO regression model. **B** SVM-RFE model. CV: cross-validate. **C** Venn diagram of two algorithms for screening key targets. **D** Columnar plot for diagnosing the risk of MDD at key targets

Machine learning algorithms were used to further explore the key targets of MDD. We performed feature screening by building LASSO regression models, and seven genes were identified as signature genes for MDD (Fig. 6A). Meanwhile, we used the SVM-RFE algorithm to evaluate the signature genes for MDD, and eight genes were identified as optimal signature genes (Fig. 6B). Five genes (AKT1, MAPK1, MYC, EGF, HSP90AA1) were obtained by intersecting the signature genes obtained from both the LASSO and the SVM-RFE models and were identified as key targets of HP for subsequent analysis (Fig. 6C). A nomogram for the risk assessment of MDD was created based on the Rms package (Fig. 6D) to exemplify the clinical practical value of the model.

### Correlation of key targets, MDD expression characterization, and gene set enrichment analysis

**Fig. 7:**
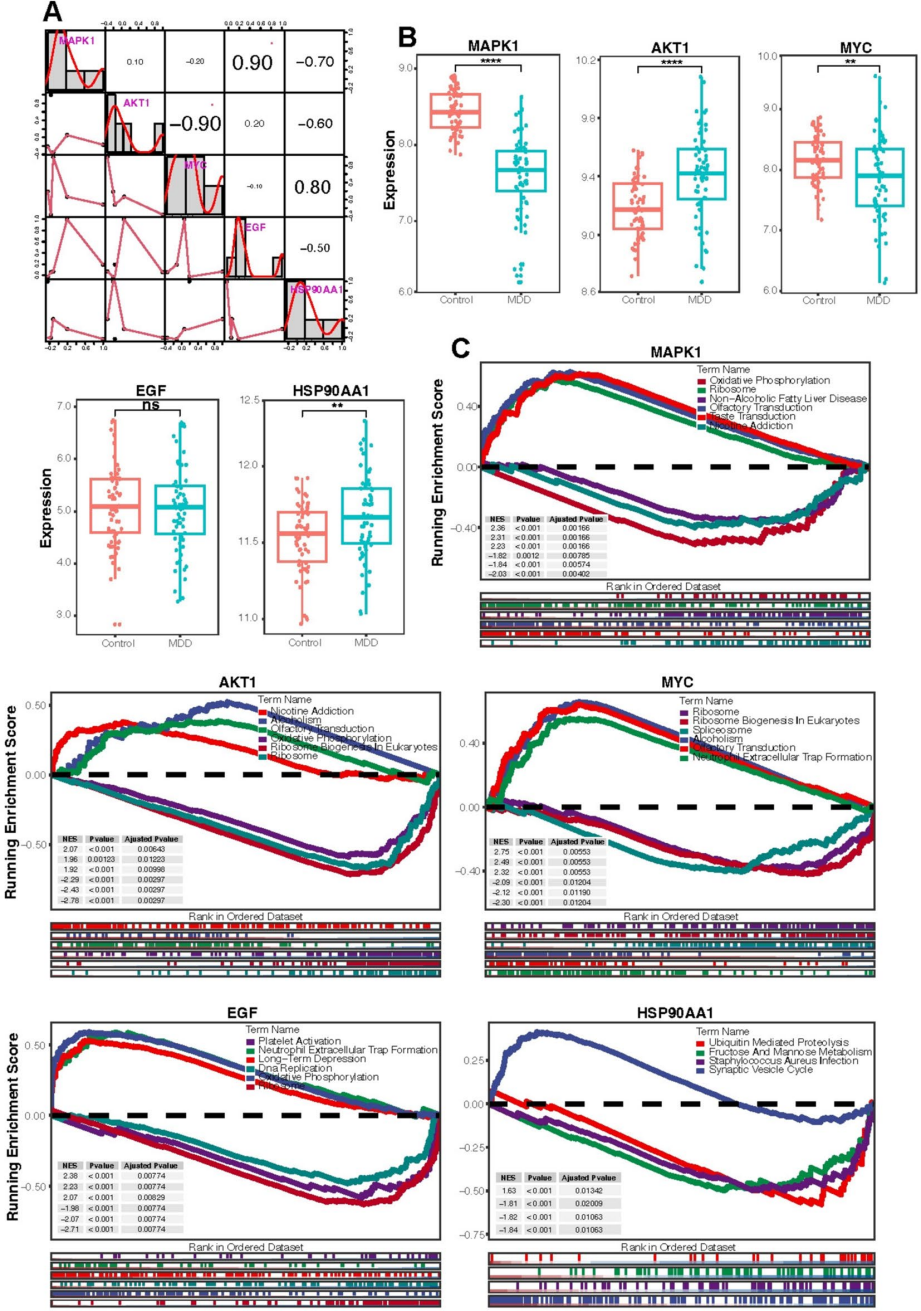
Correlation analysis of key targets, expression characterization, and GSEA enrichment results. **A** Correlation heat map of key targets, binary scatter plot with fitted lines on the left and correlation coefficients on the right. **B** Expression of key genes in blood RNA microarray data, pink is the healthy group and cyan is the MDD group. **C** GSEA enrichment results for each key gene

In order to explore the association between the five key targets, a correlation analysis was performed (Fig. 7A). The results showed that the expression of HSP90AA1 was significantly positively correlated with the expression of MYC and negatively correlated with the expression of the other three key targets. EGF was significantly positively correlated with the expression level of MAPK1 (correlation coefficient of 0.9) but MYC was significantly negatively correlated with the expression level of AKT1 (correlation coefficient of − 0.9).

We then further investigated the role of AKT1, MAPK1, MYC, EGF, and HSP90AA1 in MDD and looked at their expression profiles between the normal and MDD groups, respectively (Fig. 7B). Among them, the expression of MAPK1 and MYC was significantly higher in the normal group than in the MDD group, and the expression of AKT1 and HSP90AA1 was significantly higher in the MDD group than in the normal group, but there was no significant difference in EGF. In addition, we searched the GeneCards database for MDD-related genes to reconfirm the relationship between the five key targets and MDD, with scores of 50, 49, 49, 49, 49, 49, 46 for AKT1, MAPK1, MYC, EGF, and HSP90AA1, respectively. (N = 10,119, Median = 39, Mean = 36.04, SD = 10.28).

Subsequently, we performed a GSEA functional analysis of these five key targets and identified multiple associated pathways such as neutrophil extracellular trap formation, nicotine addiction, alcoholism, synaptic vesicle cycle, and long-term depression (Fig. 7C). Of these, we speculate that inflammation is also implicated in the development of MDD based on the enrichment of neutrophil extracellular trap formation, S. aureus infection, and other pathways.

### Immune cell level analysis

**Fig. 8:**
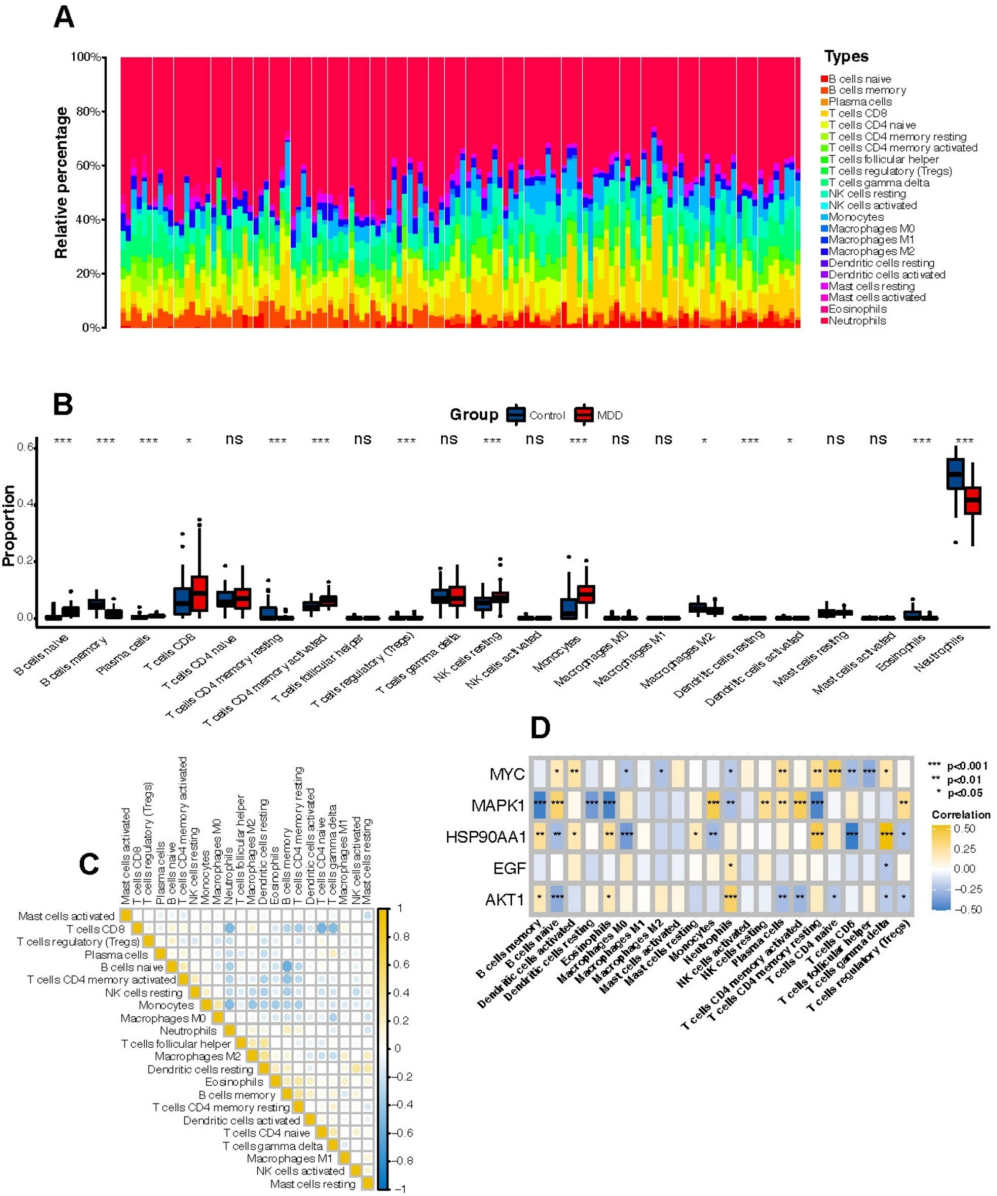
Immune cell analysis. **A** Relative proportions of 22 immune cell subpopulations in all samples in blood samples. **B** Differences in the levels of 22 immune cell types in the normal and MDD groups. (*:P < 0.05, **:P < 0.01, ***:P < 0.001, ns:no significance). **C** Correlation between the 22 immune cell subpopulations. **D** Correlation between key targets and 22 immune cell subpopulations

To explore the differential immune landscapes between the normal and MDD cohorts, we employed a robust deconvolution algorithm CIBERSORT to analyze the immune cell composition from collected samples (Fig. 8A). This advanced computational approach enabled us to accurately estimate the proportions of 22 distinct immune cell types. Subsequent comparative analysis revealed significant variations in specific immune cell populations between the two groups (Fig. 8B). Notably, neutrophils and memory B cells exhibited elevated levels in the normal group compared to the MDD group, whereas CD8T cells, naive B cells, and monocytes were significantly reduced.

The differing levels of immune cells between the normal group and the MDD group could indicate several key aspects of how the immune system may be involved in the pathophysiology of MDD: (1) Altered Immune Response: The elevated levels of neutrophils and memory B cells in the normal group suggest a more robust or active immune response compared to the MDD group. Neutrophils are crucial for rapid response to infection and inflammation, indicating a potentially more effective innate immune function in individuals without MDD. Memory B cells, part of the adaptive immune system, suggest enhanced long-term immune memory in the normal group. (2) Immune Suppression in MDD: The reduction in CD8T cells, naive B cells, and monocytes in the MDD group might reflect an immune-suppressed state or dysregulation. CD8T cells are vital for targeting and destroying infected or cancerous cells, naive B cells are essential for generating new immune responses, and monocytes are important for inflammation and tissue repair. Their decreased levels could imply a compromised ability to initiate and maintain effective immune responses in MDD patients, potentially making them more susceptible to infections or having a less effective inflammatory response, which could influence mood or neurological function. (3) Inflammation and MDD: While typically associated with active immune response, neutrophils and other cellular components could play dual roles. In MDD, altered levels of these cells could be linked to chronic inflammation, which is often observed in depressive disorders. Chronic inflammation has been hypothesized to affect brain function and mood regulation, potentially contributing to the symptoms of MDD. (4) Immune System Dysregulation: The distinct profiles of immune cells between the two groups highlight a dysregulation in the immune system associated with MDD. This dysregulation could be a contributing factor to the development or exacerbation of depressive symptoms through mechanisms such as altered cytokine production, affecting neurotransmitter systems, and impacting neuroplasticity. These findings encourage further investigation into the specific roles of these immune cells in MDD and how modulation of these cells might serve as potential therapeutic targets for treating depression, focusing on restoring normal immune function as a means to improve clinical outcomes for patients with MDD.

To further understand the functional implications of these disparities, we performed a correlation analysis to identify potential inter-cellular relationships and their impact on the immunoinflammatory pathways involved in MDD (Fig. 8C). Our findings indicated a negative correlation between neutrophils and both CD8T cells and monocytes, suggesting a possible competitive interaction or differential regulation of these cell types in the immune response to depression. Similarly, memory B cells showed a negative correlation with naive B cells, potentially reflecting a shift in B cell lineage commitment influenced by the disease state or its associated inflammatory milieu. These correlations underscore complex immune dynamics that may contribute to the pathophysiology of MDD, emphasizing the need for further investigation into how these specific immune cell alterations influence broader immunoinflammatory pathways. By elucidating these mechanisms, our study not only sheds light on the immunological underpinnings of MDD but also opens avenues for targeted immunomodulatory therapies.

In addition, we analyzed the correlation between each of the key targets and immune cells (Fig. 8D). MYC showed a significant positive correlation with CD4 naïve T cells. MAPK1 showed a significant positive correlation with naïve B cells, monocytes, and activated CD4 memory T cells, and a significant negative correlation with memory B cells, eosinophils, and resting CD4 memory T cells. HSP90AA1 showed a significant positive correlation with activated CD4 memory T cells, T HSP90AA1 was significantly positively correlated with activated CD4 memory T cells, T cells gamma delta, and negatively correlated with CD8T cells, M0 polarized macrophages. EGF was not significantly correlated with immune cells. AKT1 was significantly positively correlated with neutrophils and negatively correlated with naive B cells.

The positive correlation between MYC and CD4 naive T cells suggests that MYC, a crucial regulator of cell growth and proliferation, might be influencing the development or activation of these T cells. This could be important in diseases where T cell function is altered, such as autoimmune diseases or cancers.

MAPK1, involved in transmitting chemical signals from the outside of a cell to the inside, shows complex interactions: its positive correlation with naive B cells, monocytes, and activated CD4 memory T cells suggests a role in promoting immune responses, as these cells are key in initiating and regulating inflammation and adaptive immunity. Its negative correlation with memory B cells, eosinophils, and resting CD4 memory T cells might indicate a suppressive or regulatory role in certain immune functions, potentially influencing allergic reactions or memory responses.

HSP90AA1 is a molecular chaperone, that is important in protein folding and protecting cells under stress, its positive correlations with activated CD4 memory T cells and T cells gamma delta, suggest a role in supporting active immune responses, particularly those involving adaptive and innate-like lymphocyte functions. Its negative correlation with CD8T cells and M0 macrophages might reflect a regulatory mechanism where HSP90AA1 influences T cell cytotoxic activities and macrophage polarization, impacting inflammation and immune surveillance.

The lack of significant correlations might indicate that Epidermal Growth Factor (EGF) predominantly affects tissues via mechanisms independent of direct modulation of immune cells, or that its primary roles are non-immunological, such as tissue growth and repair.

AKT1 is kinase that pivotal in many signaling pathways including cell survival and proliferation. Its positive correlation with neutrophils suggests a role in promoting neutrophil survival or activation, potentially impacting inflammatory responses. While its negative correlation with naive B cells might indicate a suppressive effect on the maturation or activation of B cells, affecting the adaptive immune response.

These patterns of correlation can help to understand how molecular signaling pathways interact with the immune system, potentially identifying targets for therapeutic intervention in MDD where immune regulation or dysfunction is a feature. Each correlation can shed light on the potential regulatory mechanisms at play and guide further research into their biological implications in MDD.

### Regulatory mechanisms of key targets

**Fig. 9:**
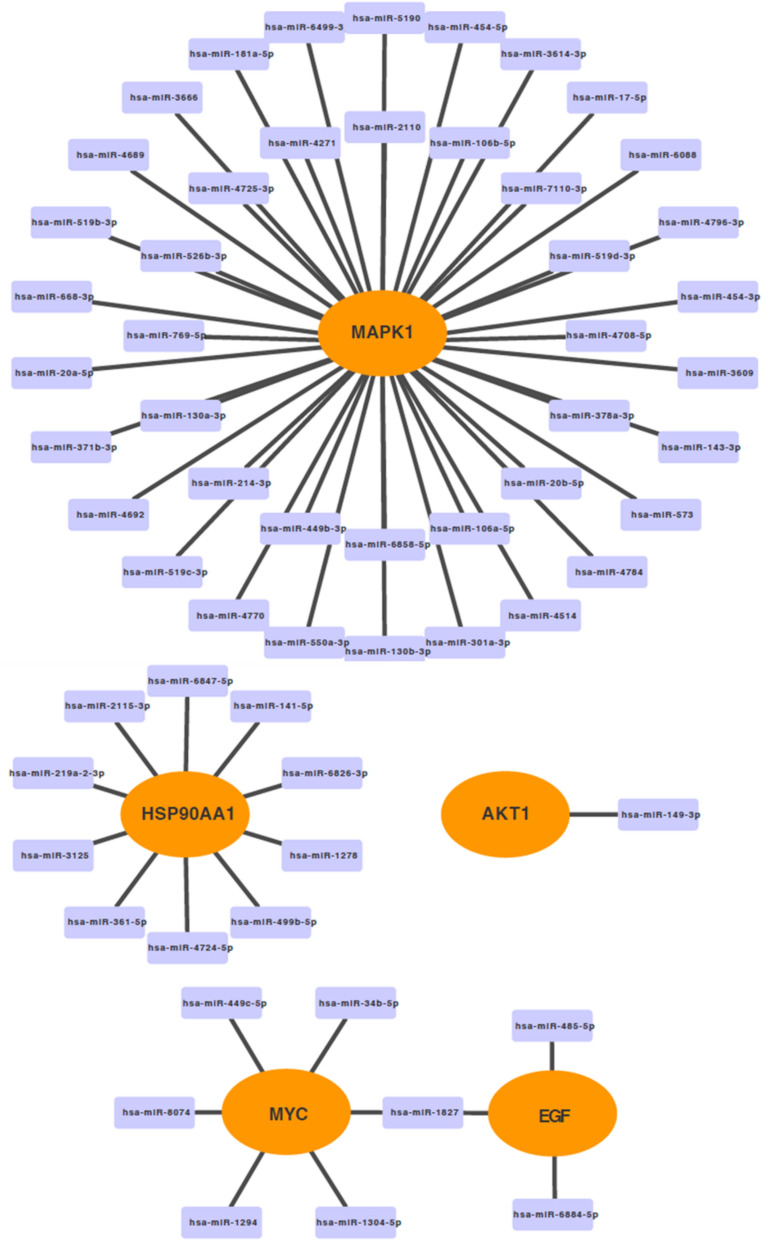
miRNA prediction of key targets. miRNA-mRNA regulatory network, rectangles indicate predicted miRNAs and ovals indicate mRNAs

A combination of 6 databases was used to make miRNA predictions for each key target and to construct the miRNA-mRNA regulatory network (Fig. 9). 42, 10, 6, 3, and 1 miRNAs were predicted for MAPK1, HSP90AA1, MYC, EGF, and AKT1 respectively, with hsa-miR-1827 being the common miRNA predicted for EGF and MYC. Detailed results are available in S-Tables 2.

### Molecular docking verification

**Fig. 10:**
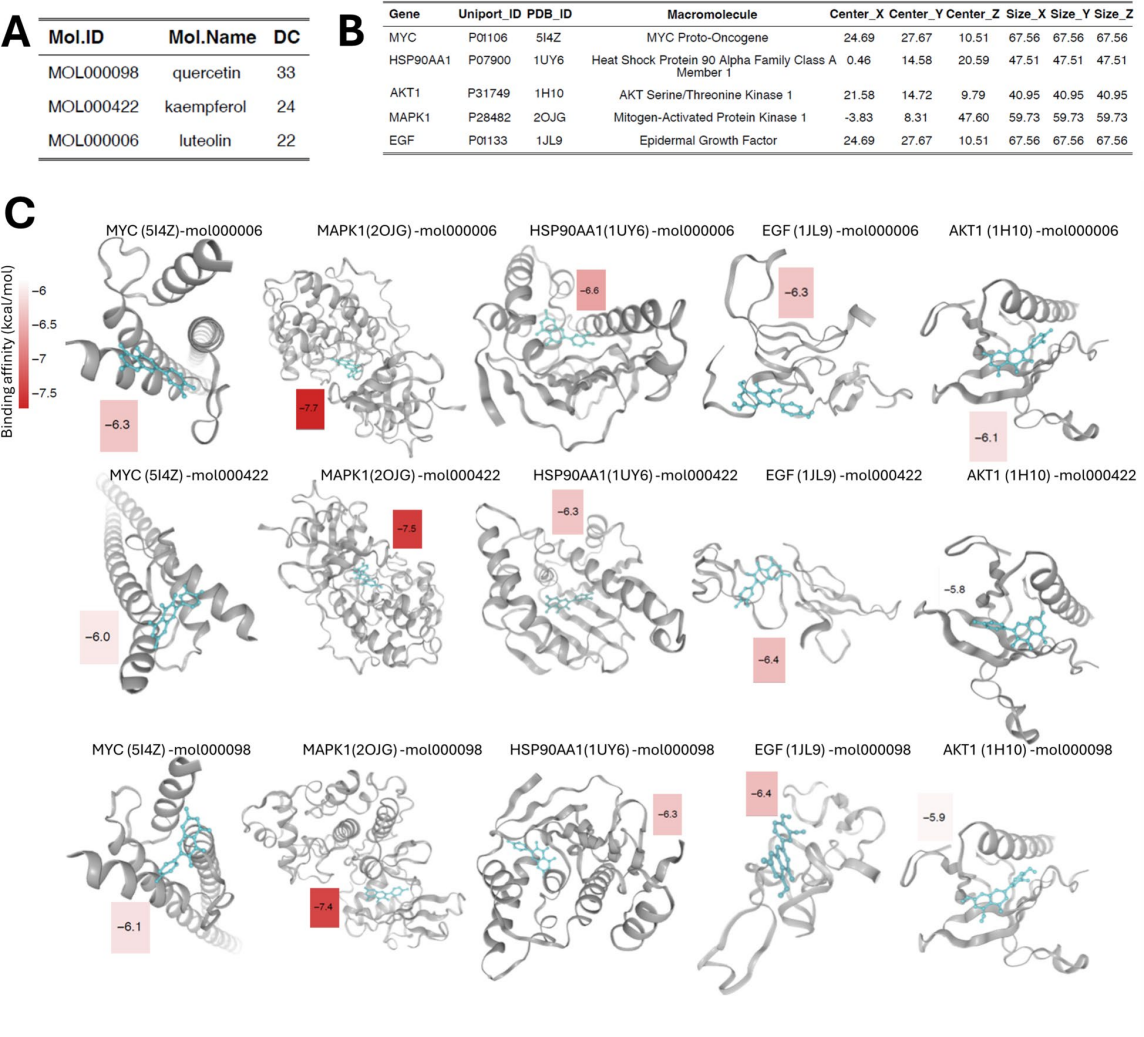
Molecular docking verification. **A** Active ingredients of HP selected for docking analysis, DC is the Degree Centrality of the node in the “Component-Target-Pathway” network. **B** Key targets selected protein IDs and docking pockets. **C** Visualization of docking models with binding affinity. The PDB IDs are shown in parentheses

To predict the potential interaction effects of HP on these five key targets in MDD, we used three major active ingredients of HP, including quercetin, kaempferol, and luteolin, to dock with MAPK1, EGF, HSP90AA1, AKT1, and MYC, respectively (Fig. 10A, B). Each pair of docking was conducted nine times (detailed results are provided in S-Table 3) and the lowest binding affinity was recorded. The docking of any of the active ingredients and the five key targets resulted in the binding affinity of − 7.7 to − 5.8, which was less than − 5 kcal/mol, demonstrating that HP might have good binding ability to the key targets in MDD patients (Fig. 10C). All of the resulting models are presented in Fig. 10D.

## Discussion

Depression is a prevalent and difficult-to-treat global psychiatric disorder, with depressed mood and unresponsiveness as typical symptoms, causing serious disturbance to the life and work of patients [50]. *Hypericum* *perforatum* (HP) has good antidepressant properties and its efficacy has been confirmed in several clinical studies. Although the antidepressant effect of HP has been well studied, the mechanism is not fully understood [51, 52]. Combining biological and pharmacological principles, drugs do not directly target disease-related genes, but they can also be regulated by upstream and downstream molecules [53].

Bioinformatic analysis and databases have been wildly applied to understand human disease and contribute to the development of disease diagnostic and prognostics [54–67]. Our study emphasizes the importance of “drug target-disease target” in the “component-target-pathway” approach by combining the KEGG pathway to retrieve all potential biomarkers associated with Kanye Goldenseal and MDD, and by using machine learning to screen and Identify MAPK1, EGF, HSP90AA1, AKT1, MYC as key targets for HP treatment of MDD.

In this study, we intersected 933 MDD-related potential targets from differentially expressed genes and WGCNA key module genes, 982 predicted MDD targets from three disease databases, and 275 HP active ingredient targets for KEGG pathway enrichment analysis to obtain KEGGa, KEGGb, and KEGGc. These results corroborated each other and effectively reduced the probability of false positives. The results show that the main pathways are Fluid shear stress and atherosclerosis, NOD-like receptor signaling pathway, and C-type lectin receptor signaling pathway. Different fluid shear forces affect the expression of endothelial cell genes, and according to existing studies, endothelial cell dysfunction can exacerbate cardiovascular diseases such as atherosclerosis [68, 69]. Moreover, depression leads to sympathetic hyperactivity in the body. Platelet activation also leads to an increased risk of arrhythmias, which over time can lead to damage to small blood vessels and microvessels, increasing the prevalence of cardiovascular disease [70–72]. Several studies have linked inflammatory vesicles to depression. NLRP1, NLRP2, and NLRP3 are all members of the NLR family. They are expressed on microglia and astrocytes as inflammatory vesicles and when activated produce inflammatory factors leading to neuroinflammation and thus depressive symptoms [73, 74]. In addition, it has been suggested that C-type lectin receptors accelerate nucleosome-induced oxidative stress and neuroinflammation in microglia, enhancing negative mood [75]. All of the above pathways are similar to our study, and we also note the now well-accepted neuroinflammation as a cause of endogenous depression to pave the way for subsequent analyses [10, 76, 77].

Previous studies have often emphasized the role of drug molecules in the regulation of a small number of proteins [78, 79]. However, individual protein molecules are only one member of an interplay network, and it is a range of signal transduction and protein interregulation that really affects biological response pairs. In addition to acting directly on MDD targets, drugs can also act first on certain genes and indirectly regulate MDD through the proteins encoded by these genes. In order to collect the genes that are direct and indirect targets of drug action, all genes in 13 key pathways were cross-analyzed with the active ingredient targets of Onychomycetes to generate 76 potential therapeutic targets.

Subsequently, we constructed a “component-target-pathway” network and calculated 22 core network genes using the MCODE plug-in. Finally, AKT1, MAPK1, MYC, EGF, and HSP90AA1 in peripheral blood were identified as key targets for HP treatment of MDD by SVM-RFE and LASSO algorithms. The scores of these five key targets were searched in the GeneCards database and each was higher than Median + SD and Mean + SD for all genes. Combined with the column line graph model and ROC results, AKT1, MAPK1, MYC, EGF, and HSP90AA1 have the potential as key therapeutic targets for the diagnosis of MDD [80]. A GSEA analysis of the five key therapeutic targets was performed. Alcoholism is mainly manifested in dependence on alcohol, which depletes OMEGA-3 and disrupts normal levels of dopamine and serotonin [81, 82]. It also depletes vitamin B6, which converts tryptophan into serotonin, thus making people more prone to anxiety and depression. Nicotine can cause the brain to release dopamine to temporarily improve mood, but nicotine can create tolerance and addiction [83]. Some studies have shown that the severity and recurrence of depression increase as smoking rates increase [84]. Interestingly, we note the Neutrophil Extracellular Trap Formation, where several studies have suggested that “depression is accompanied by immunosuppression” and that neutrophils can release pro-inflammatory cytokines and neutrophil extracellular traps (NETs) that induce endothelial dysfunction, further recruiting inflammatory cells and promoting neurological inflammation and enhance depressive symptoms [85, 86].

A number of early studies have demonstrated the antidepressant efficacy of HP, being used in mild and moderate depression, with significant effects in adolescents and a significant reduction in side effects [87–89]. One of the main antidepressant compounds considered to be active is Hyperforin, which has been shown to indirectly inhibit the reuptake of 5-hydroxytryptamine. It inhibits the reuptake of dopamine, glutamate, norepinephrine, and gamma-aminobutyric acid (GABA) by competitively affecting the activity of transporter proteins, thus achieving an antidepressant effect [21, 90]. But in addition to neurotransmitters, immune inflammation should also be taken into account [8]. In an immune cell analysis, we found that depressed patients had higher expression of naive B cells, CD8T cells, CD4-activated memory T cells, resting NK cells, and monocytes in the peripheral blood than in the normal group [91, 92]. There are a number of studies and mouse models that confirm that depressed patients have reduced circulating T cells and regulatory B cells [93]. Secondly, studies have also mentioned that premature senescence of monocytes in MDD patients increases a more severe inflammatory response, particularly in patients over 28 years of age [94]. Quercetin may inhibit the function of TNF-α and other types of cytokines to reduce neuroinflammation and alleviate symptoms of depression [74]. Biologically and pharmacologically, quercetin may also help to accelerate the degradation of neurotransmitters in the synaptic gap and help serotonin and dopamine to work better [95]. Kaempferol increases the AKT/β-linked protein cascade in the prefrontal cortex and reduces inflammatory factor levels [96, 97]. Lignocaine reduces IL-6 production by astrocytes, decreases serum levels of IL-6, TNF-α, and corticosterone, and increases mature brain-derived neurotrophic factor (BDNF), dopamine, and norepinephrine levels to exert antidepressant effects [98, 99]. Furthermore, and most notably, the neutrophil normal group was significantly higher than the MDD group, defying the norm of the immunoinflammatory doctrine. With the same data set, we found some studies where the neutrophil MDD group was higher than the normal group, and some studies where the normal group was higher than the MDD group [100]. In our previous analysis there is a pathway called “neutrophil extracellular trap formation”, where the activation of NETosis leads to neutrophil death through apoptosis or necrosis, and patients with MDD have a stronger inflammatory response in the peripheral blood than the normal group, so the presence of suicidal NETosis makes the neutrophil percentage in MDD patients less than normal. The presence of suicidal NETosis resulted in a smaller proportion of neutrophils in MDD patients than in the normal group [101]. However, because the formation of NETs is a dynamic equilibrium, relevant reports are scarce and need to be further explored in future studies.

The five key genes identified—AKT1, MAPK1, MYC, EGF, and HSP90AA1—are all significant in various cellular processes including growth, survival, and proliferation, which are pivotal in numerous diseases, including MDD. AKT1 is a crucial player in the PI3K/Akt signaling pathway, involved in cell survival, proliferation, and metabolism [102]. Dysregulation of this pathway has been associated with impaired neuronal survival and synaptic plasticity, which are considered central mechanisms in the pathology of MDD. Alterations in AKT1 activity can affect brain-derived neurotrophic factor (BDNF) levels, impacting mood regulation and response to stress [103]. Role in Cellular Function: MAPK1 is part of the MAP kinase signaling pathway, involved in transmitting chemical signals from the cell surface to the DNA in the nucleus [104]. This gene plays a role in neuronal plasticity, survival, and differentiation. Abnormal MAPK1 signaling has been implicated in impaired stress response and neuroplasticity, which are key features of MDD [104]. MYC is a regulator gene that codes for a transcription factor. It is involved in cell cycle progression, apoptosis, and cellular transformation [105, 106]. Although more traditionally linked with cancer, MYC is also important in brain development and function [106]. Overexpression of MYC has been suggested to influence brain cell metabolism and survival [107], potentially contributing to the neurobiological changes seen in MDD. EGF is a growth factor that stimulates cell growth, proliferation, and differentiation by binding to its receptor, EGFR [108]. EGF has been shown to promote neurogenesis and is involved in the neuroendocrine response to stress [109, 110]. Altered levels of EGF and disruptions in its signaling pathways have been observed in depressive disorders, suggesting its role in the pathophysiology of MDD. HSP90AA1 is the heat shock protein 90 alpha family class a member 1. This gene encodes a member of the heat shock protein 90 family, which are molecular chaperones involved in signal transduction, protein folding, and degradation [111]. HSP90AA1 helps in the proper folding of proteins and protection against cellular stress [111]. Dysregulation of HSP90AA1 can lead to impaired stress response and cellular resilience, factors that are potentially linked to the development and severity of MDD. Each of these genes, through their respective roles in signaling pathways, neuroplasticity, and stress response, could contribute to the pathophysiology of MDD, making them interesting targets for further research and potentially therapeutic intervention.

The diagnosis and prognosis of miRNAs have been demonstrated for various types of cancer, and miRNAs are important regulators of epigenetic mechanisms [112]. The field of psychiatry is also placing increasing emphasis on the relationship between miRNA expression and the regulation of proteins [113]. We predicted the corresponding miRNAs based on five key targets, with a total of 42 miRNAs predicted for MAPK1 and has-miR-1827 synergistically regulating MYC and EGF [114]. In the GeneCards database, we retrieved MDD-related scores for the five key targets and counted them, showing that they are all closely associated with MDD. Finally, we used molecular docking to predict the interactions between HP and AKT1, MAPK1, MYC, EGF, and HSP90AA1. The strongest binding here was MAPK1, and linkage to the miRNA prediction network suggests that MAPK1 can be explored in depth as a potential target for HP in the treatment of MDD.

Immunoinflammatory has been reported important for many human diseases [115–118]. Most of the previous research has been conducted using the neurotransmitter theory, and the immunoinflammatory theory has only recently been applied to the study of depression. *Hypericum* *perforatum*, also known as St. John's wort, has been shown in many studies to improve symptoms of depression, even more so than some placebos. But again it is still not on the list of standard antidepressants, so more research is needed to support this. This study adopts a new way of thinking to explore MDD from a pathway and immune-inflammatory perspective, combining WGCNA, protein interaction networks, machine learning, and molecular docking to elucidate the mechanism of action of HP for MDD. The study further identified AKT1, MAPK1, MYC, EGF, and HSP90AA1 as key targets for the treatment of MDD by HP. This study still has some limitations, and the mechanism of action of neutrophils in MDD patients needs to be validated in conjunction with animal experiments to explore the balance between the occurrence of suicidal NETs and the immune role it plays.

We acknowledge the limitations posed by the disproportionate female-to-male ratio of 3:1 in our study cohort. This gender distribution reflects the higher prevalence of MDD among females compared to males, as reported in epidemiological studies [119–121]. However, this imbalance may also limit the generalizability of our findings across genders. To understand the potential impact of this gender bias, it is important to consider the biological and hormonal differences between genders that could influence the pharmacodynamics and pharmacokinetics of natural products from HP. For instance, differences in hormone levels, such as estrogen, might modulate the bioactivity of HP compounds differently in males and females [122], potentially affecting the efficacy and safety profiles. Further studies with a separate gender and balanced gender distribution are required to validate our findings and ensure they are applicable to either gender or both males and females. Additionally, subgroup analyses focusing on gender-specific responses to HP treatment could provide deeper insights into the molecular mechanisms underlying the gender differences observed in MDD response rates.

Another limitation is no external validation. We have reviewed previously published data and found that the dataset we analyzed is unique in that it includes MDD signals in blood, presenting a challenge for external validation, as comparable datasets are not available. To mitigate potential biases and avoid overfitting, we employed multiple methodological approaches. First, we utilized the LASSO regression model (a method that was wildly used in previous studies [61, 63, 67]), which features a shrinkage path for the coefficients, with the regularization parameter λ selected through cross-validation. The AUC curve displayed below the shrinkage path graphically illustrates the model's performance across varying λ values, enabling the selection of an optimal λ, generally near the AUC curve’s peak, to ensure that the model neither overfits nor underfits. Secondly, we implemented tenfold cross-validation in our analysis, particularly in the SVM-RFE model. This technique is instrumental in evaluating the model's stability and generalization capacity by iteratively splitting the data into training and test sets, thus assessing the model's efficacy across different subsets. Such a method combinding LASSO and tenfold cross-validation in SVM-RFE model not only aids in preventing overfitting but also assures robust predictive accuracy on unseen data.

Overfitting remains a pervasive concern in machine learning models, particularly given the constraints posed by relatively small sample sizes. In scenarios where the dataset is limited, the probability that the model will learn noise rather than underlying data patterns increases significantly, compromising the model's ability to generalize to new data. This issue is of particular importance in our study, where the modest sample size heightens the risk of overfitting. In this study, the cohort consisted of 128 samples. Ideally, the number of samples should considerably surpass the number of features to ensure that the model discerns genuine patterns rather than noise, thus minimizing the risk of overfitting. A close ratio between the number of samples and features may lead to overfitting, which could degrade the model’s performance on new, unseen data [123]. It is generally accepted that having more samples than features helps prevent this issue. In our analysis, the ratio of 128 samples to 22 genetic variables is reasonably balanced—not overly constrained but adequately stringent. Furthermore, to enhance model stability and reduce overfitting, we implemented tenfold cross-validation. This method involves repetitively partitioning the dataset into distinct training and validation sets, which is crucial for verifying the model's robustness across different subsets of data.

## Conclusions

In summary, we have used bioinformatic methods to identify and validate key targets of HP for the treatment of MDD, and then investigated the mechanism of action of HP for MDD through signaling pathways and immune inflammation, identifying AKT1, MAPK1, MYC, EGF, and HSP90AA1 as key therapeutic target genes, which provide a theoretical basis for clinical treatment and drug development.

## Supplementary Information

Supplementary Material 1.

Supplementary Material 2.

Supplementary Material 3.

## Data Availability

The microarray data were previously published as GSE98793 [29] in the GEO database (https://www.ncbi.nlm.nih.gov/geo/). Other original data presented in the study are included in the article/Supplementary Material, further inquiries can be directed to the corresponding authors.

## References

[1] Williams JW Jr, Mulrow CD, Chiquette E, Noël PH, Aguilar C, Cornell J. A systematic review of newer pharmacotherapies for depression in adults: evidence report summary. Ann Intern Med. 2000;132:743–56. 10.7326/0003-4819-132-9-200005020-00011. 10787370 10.7326/0003-4819-132-9-200005020-00011

[2] Kato M, Hori H, Inoue T, Iga J, Iwata M, Inagaki T, Shinohara K, Imai H, Murata A, Mishima K, et al. Discontinuation of antidepressants after remission with antidepressant medication in major depressive disorder: a systematic review and meta-analysis. Mol Psychiatry. 2021;26:118–33. 10.1038/s41380-020-0843-0. 32704061 10.1038/s41380-020-0843-0 PMC7815511

[3] Kupfer DJ, Frank E, Phillips ML. Major depressive disorder: new clinical, neurobiological, and treatment perspectives. Lancet (London, England). 2012;379:1045–55. 10.1016/s0140-6736(11)60602-8. 22189047 10.1016/S0140-6736(11)60602-8 PMC3397431

[4] Gadad BS, Jha MK, Czysz A, Furman JL, Mayes TL, Emslie MP, Trivedi MH. Peripheral biomarkers of major depression and antidepressant treatment response: current knowledge and future outlooks. J Affect Disord. 2018;233:3–14. 10.1016/j.jad.2017.07.001. 28709695 10.1016/j.jad.2017.07.001 PMC5815949

[5] Belujon P, Grace AA. Dopamine system dysregulation in major depressive disorders. Int J Neuropsychopharmacol. 2017;20:1036–46. 10.1093/ijnp/pyx056. 29106542 10.1093/ijnp/pyx056 PMC5716179

[6] Jin Y, Cui R, Zhao L, Fan J, Li B. Mechanisms of *Panax**ginseng* action as an antidepressant. Cell Prolif. 2019;52: e12696. 10.1111/cpr.12696. 31599060 10.1111/cpr.12696 PMC6869450

[7] Tartt AN, Mariani MB, Hen R, Mann JJ, Boldrini M. Dysregulation of adult hippocampal neuroplasticity in major depression: pathogenesis and therapeutic implications. Mol Psychiatry. 2022;27:2689–99. 10.1038/s41380-022-01520-y. 35354926 10.1038/s41380-022-01520-y PMC9167750

[8] Beurel E, Toups M, Nemeroff CB. The bidirectional relationship of depression and inflammation: double trouble. Neuron. 2020;107:234–56. 10.1016/j.neuron.2020.06.002. 32553197 10.1016/j.neuron.2020.06.002 PMC7381373

[9] Miller AH, Maletic V, Raison CL. Inflammation and its discontents: the role of cytokines in the pathophysiology of major depression. Biol Psychiat. 2009;65:732–41. 10.1016/j.biopsych.2008.11.029. 19150053 10.1016/j.biopsych.2008.11.029 PMC2680424

[10] Milaneschi Y, Allers KA, Beekman ATF, Giltay EJ, Keller S, Schoevers RA, Süssmuth SD, Niessen HG, Penninx B. The association between plasma tryptophan catabolites and depression: the role of symptom profiles and inflammation. Brain Behav Immun. 2021;97:167–75. 10.1016/j.bbi.2021.07.007. 34252568 10.1016/j.bbi.2021.07.007

[11] Haixia W, Shu M, Li Y, Panpan W, Kehuan S, Yingquan X, Hengrui L, Xiaoguang L, Zhidi W, Ling O. Effectiveness associated with different therapies for senile osteoporosis: a network meta-analysis. J Trad Chin Med. 2020;40:17–27. 32227762

[12] Hengrui L. An example of toxic medicine used in Traditional Chinese Medicine for cancer treatment. J Tradit Chin Med. 2023;43:209. 36994507 10.19852/j.cnki.jtcm.2023.02.001 PMC10012187

[13] Liu H, Xiong Y, Wang H, Yang L, Wang C, Liu X, Wu Z, Li X, Ou L, Zhang R. Effects of water extract from epimedium on neuropeptide signaling in an ovariectomized osteoporosis rat model. J Ethnopharmacol. 2018;221:126–36. 29705515 10.1016/j.jep.2018.04.035

[14] Liu H, Xiong Y, Zhu X, Gao H, Yin S, Wang J, Chen G, Wang C, Xiang L, Wang P. Icariin improves osteoporosis, inhibits the expression of PPARγ, C/EBPα, FABP4 mRNA, N1ICD and jagged1 proteins, and increases Notch2 mRNA in ovariectomized rats. Exp Ther Med. 2017;13:1360–8. 28413478 10.3892/etm.2017.4128 PMC5377361

[15] Ou L, Liu HR, Shi XY, Peng C, Zou YJ, Jia JW, Li H, Zhu ZX, Wang YH, Su BM, et al. *Terminalia**chebula* Retz. aqueous extract inhibits the *Helicobacter**pylori*-induced inflammatory response by regulating the inflammasome signaling and ER-stress pathway. J Ethnopharmacol. 2024;320: 117428. 10.1016/j.jep.2023.117428. 37981121 10.1016/j.jep.2023.117428

[16] Ou L, Zhu Z, Hao Y, Li Q, Liu H, Chen Q, Peng C, Zhang C, Zou Y, Jia J, et al. 1,3,6-Trigalloylglucose: a novel potent anti-*Helicobacter**pylori* adhesion agent derived from aqueous extracts of *Terminalia**chebula* Retz. Molecules. 2024;29:1161. 38474673 10.3390/molecules29051161 PMC10935070

[17] Peng C, Feng Z, Ou L, Zou Y, Sang S, Liu H, Zhu W, Gan G, Zhang G, Yao M. Syzygium aromaticum enhances innate immunity by triggering macrophage M1 polarization and alleviates *Helicobacter**pylori*-induced inflammation. J Funct Foods. 2023;107: 105626.

[18] Wu Z, Ou L, Wang C, Yang L, Wang P, Liu H, Xiong Y, Sun K, Zhang R, Zhu X. Icaritin induces MC3T3-E1 subclone14 cell differentiation through estrogen receptor-mediated ERK1/2 and p38 signaling activation. Biomed Pharmacother. 2017;94:1–9. 28742995 10.1016/j.biopha.2017.07.071

[19] Hengrui L. Toxic medicine used in Traditional Chinese Medicine for cancer treatment: are ion channels involved? J Tradit Chin Med. 2022;42:1019. 36378062 10.19852/j.cnki.jtcm.20220815.005 PMC9924727

[20] Linde K, Mulrow CD, Berner M, Egger M. St John’s wort for depression. Cochrane Database Syst Rev. 2005. 10.1002/14651858.CD000448.pub2. 15846605 10.1002/14651858.CD000448.pub2

[21] Linde K, Berner MM, Kriston L. St John’s wort for major depression. Cochrane Database Syst Rev. 2008. 10.1002/14651858.CD000448.pub3. 18843608 10.1002/14651858.CD000448.pub3 PMC7032678

[22] Davis AP, Wiegers TC, Wiegers J, Wyatt B, Johnson RJ, Sciaky D, Barkalow F, Strong M, Planchart A, Mattingly CJ. CTD tetramers: a new online tool that computationally links curated chemicals, genes, phenotypes, and diseases to inform molecular mechanisms for environmental health. Toxicol Sci. 2023;195:155–68. 37486259 10.1093/toxsci/kfad069 PMC10535784

[23] Piñero J, Ramírez-Anguita JM, Saüch-Pitarch J, Ronzano F, Centeno E, Sanz F, Furlong LI. The DisGeNET knowledge platform for disease genomics: 2019 update. Nucleic Acids Res. 2020;48:D845–55. 31680165 10.1093/nar/gkz1021 PMC7145631

[24] Stelzer G, Rosen N, Plaschkes I, Zimmerman S, Twik M, Fishilevich S, Stein TI, Nudel R, Lieder I, Mazor Y. The GeneCards suite: from gene data mining to disease genome sequence analyses. Curr Protoc Bioinf. 2016;54:1.30.31-31.30.33. 10.1002/cpbi.5 27322403

[25] Ru J, Li P, Wang J, Zhou W, Li B, Huang C, Li P, Guo Z, Tao W, Yang Y, et al. TCMSP: a database of systems pharmacology for drug discovery from herbal medicines. J Cheminf. 2014;6:13. 10.1186/1758-2946-6-13. 10.1186/1758-2946-6-13 PMC4001360 24735618

[26] Bader GD, Hogue CW. An automated method for finding molecular complexes in large protein interaction networks. BMC Bioinform. 2003;4:2. 10.1186/1471-2105-4-2. 10.1186/1471-2105-4-2 PMC149346 12525261

[27] Friedman J, Hastie T, Tibshirani R. Regularization paths for generalized linear models via coordinate descent. J Stat Softw. 2010;33:1. 20808728 PMC2929880

[28] Sanz H, Valim C, Vegas E, Oller JM, Reverter F. SVM-RFE: selection and visualization of the most relevant features through non-linear kernels. BMC Bioinform. 2018;19:1–18. 10.1186/s12859-018-2451-4 PMC6245920 30453885

[29] Leday GG, Vértes PE, Richardson S, Greene JR, Regan T, Khan S, Henderson R, Freeman TC, Pariante CM, Harrison NA. Replicable and coupled changes in innate and adaptive immune gene expression in two case-control studies of blood microarrays in major depressive disorder. Biol Psychiat. 2018;83:70–80. 28688579 10.1016/j.biopsych.2017.01.021 PMC5720346

[30] Ritchie ME, Phipson B, Wu D, Hu Y, Law CW, Shi W, Smyth GK. limma powers differential expression analyses for RNA-sequencing and microarray studies. Nucleic Acids Res. 2015;43: e47. 10.1093/nar/gkv007. 25605792 10.1093/nar/gkv007 PMC4402510

[31] Langfelder P, Horvath S. WGCNA: an R package for weighted correlation network analysis. BMC Bioinform. 2008;9:559. 10.1186/1471-2105-9-559. 10.1186/1471-2105-9-559 PMC2631488 19114008

[32] Wu T, Hu E, Xu S, Chen M, Guo P, Dai Z, Feng T, Zhou L, Tang W, Zhan L, et al. clusterProfiler 4.0: a universal enrichment tool for interpreting omics data. Innovation (Cambridge (Mass)). 2021;2: 100141. 10.1016/j.xinn.2021.100141. 34557778 10.1016/j.xinn.2021.100141 PMC8454663

[33] Luo W, Brouwer C. Pathview: an R/Bioconductor package for pathway-based data integration and visualization. Bioinformatics (Oxford, England). 2013;29:1830–1. 10.1093/bioinformatics/btt285. 23740750 10.1093/bioinformatics/btt285 PMC3702256

[34] Szklarczyk D, Kirsch R, Koutrouli M, Nastou K, Mehryary F, Hachilif R, Gable AL, Fang T, Doncheva NT, Pyysalo S. The STRING database in 2023: protein–protein association networks and functional enrichment analyses for any sequenced genome of interest. Nucleic Acids Res. 2023;51:D638–46. 36370105 10.1093/nar/gkac1000 PMC9825434

[35] Doncheva NT, Morris JH, Gorodkin J, Jensen LJ. Cytoscape StringApp: network analysis and visualization of proteomics data. J Proteome Res. 2019;18:623–32. 10.1021/acs.jproteome.8b00702. 30450911 10.1021/acs.jproteome.8b00702 PMC6800166

[36] Engebretsen S, Bohlin J. Statistical predictions with glmnet. Clin Epigenet. 2019;11:123. 10.1186/s13148-019-0730-1. 10.1186/s13148-019-0730-1 PMC6708235 31443682

[37] Robin X, Turck N, Hainard A, Tiberti N, Lisacek F, Sanchez JC, Müller M. pROC: an open-source package for R and S+ to analyze and compare ROC curves. BMC Bioinform. 2011;12:77. 10.1186/1471-2105-12-77. 10.1186/1471-2105-12-77 PMC3068975 21414208

[38] Newman AM, Liu CL, Green MR, Gentles AJ, Feng W, Xu Y, Hoang CD, Diehn M, Alizadeh AA. Robust enumeration of cell subsets from tissue expression profiles. Nat Methods. 2015;12:453–7. 10.1038/nmeth.3337. 25822800 10.1038/nmeth.3337 PMC4739640

[39] Chen Y, Wang X. miRDB: an online database for prediction of functional microRNA targets. Nucleic Acids Res. 2020;48:D127–31. 31504780 10.1093/nar/gkz757 PMC6943051

[40] Sticht C, De La Torre C, Parveen A, Gretz N. miRWalk: an online resource for prediction of microRNA binding sites. PLoS ONE. 2018;13: e0206239. 30335862 10.1371/journal.pone.0206239 PMC6193719

[41] Miranda KC, Huynh T, Tay Y, Ang Y-S, Tam W-L, Thomson AM, Lim B, Rigoutsos I. A pattern-based method for the identification of MicroRNA binding sites and their corresponding heteroduplexes. Cell. 2006;126:1203–17. 16990141 10.1016/j.cell.2006.07.031

[42] Kang J, Tang Q, He J, Li L, Yang N, Yu S, Wang M, Zhang Y, Lin J, Cui T. RNAInter v4.0: RNA interactome repository with redefined confidence scoring system and improved accessibility. Nucleic Acids Res. 2022;50:D326–32. 34718726 10.1093/nar/gkab997 PMC8728132

[43] McGeary SE, Lin KS, Shi CY, Pham TM, Bisaria N, Kelley GM, Bartel DP. The biochemical basis of microRNA targeting efficacy. Science. 2019;366:eaav1741. 31806698 10.1126/science.aav1741 PMC7051167

[44] Gaillard T. Evaluation of AutoDock and AutoDock Vina on the CASF-2013 benchmark. J Chem Inf Model. 2018;58:1697–706. 10.1021/acs.jcim.8b00312. 29989806 10.1021/acs.jcim.8b00312

[45] Aronov AM, Baker C, Bemis GW, Cao J, Chen G, Ford PJ, Germann UA, Green J, Hale MR, Jacobs M. Flipped out: structure-guided design of selective pyrazolylpyrrole ERK inhibitors. J Med Chem. 2007;50:1280–7. 17300186 10.1021/jm061381f

[46] Lu H-S, Chai J-J, Li M, Huang B-R, He C-H, Bi R-C. Crystal structure of human epidermal growth factor and its dimerization. J Biol Chem. 2001;276:34913–7. 11438527 10.1074/jbc.M102874200

[47] Wright L, Barril X, Dymock B, Sheridan L, Surgenor A, Beswick M, Drysdale M, Collier A, Massey A, Davies N. Structure-activity relationships in purine-based inhibitor binding to HSP90 isoforms. Chem Biol. 2004;11:775–85. 15217611 10.1016/j.chembiol.2004.03.033

[48] Thomas CC, Deak M, Alessi DR, van Aalten DM. High-resolution structure of the pleckstrin homology domain of protein kinase b/akt bound to phosphatidylinositol (3, 4, 5)-trisphosphate. Curr Biol. 2002;12:1256–62. 12176338 10.1016/s0960-9822(02)00972-7

[49] Jung LA, Gebhardt A, Koelmel W, Ade CP, Walz S, Kuper J, von Eyss B, Letschert S, Redel C, d’Artista L. OmoMYC blunts promoter invasion by oncogenic MYC to inhibit gene expression characteristic of MYC-dependent tumors. Oncogene. 2017;36:1911–24. 27748763 10.1038/onc.2016.354

[50] Culpepper L, Lam RW, McIntyre RS. Cognitive impairment in patients with depression: awareness, assessment, and management. J Clin Psychiatry. 2017;78:1383–94. 10.4088/JCP.tk16043ah5c. 29345866 10.4088/JCP.tk16043ah5c

[51] Pu J, Liu Y, Zhang H, Tian L, Gui S, Yu Y, Chen X, Chen Y, Yang L, Ran Y, et al. An integrated meta-analysis of peripheral blood metabolites and biological functions in major depressive disorder. Mol Psychiatry. 2021;26:4265–76. 10.1038/s41380-020-0645-4. 31959849 10.1038/s41380-020-0645-4 PMC8550972

[52] Mora C, Zonca V, Riva MA, Cattaneo A. Blood biomarkers and treatment response in major depression. Expert Rev Mol Diagn. 2018;18:513–29. 10.1080/14737159.2018.1470927. 29701114 10.1080/14737159.2018.1470927

[53] Fries GR, Saldana VA, Finnstein J, Rein T. Molecular pathways of major depressive disorder converge on the synapse. Mol Psychiatry. 2022. 10.1038/s41380-022-01806-1. 36203007 10.1038/s41380-022-01806-1 PMC9540059

[54] Li Y, Liu H. Clinical powers of aminoacyl tRNA synthetase complex interacting multifunctional protein 1 (AIMP1) for head-neck squamous cell carcinoma. Cancer Biomark. 2022;34:359–74. 35068446 10.3233/CBM-210340 PMC12364190

[55] Liu H. Expression and potential immune involvement of cuproptosis in kidney renal clear cell carcinoma. Cancer Genet. 2023;274:21–5. 36963335 10.1016/j.cancergen.2023.03.002

[56] Liu H. Association between sleep duration and depression: a Mendelian randomization analysis. J Affect Disord. 2023;335:152–4. 37178827 10.1016/j.jad.2023.05.020

[57] Liu H, Dilger JP, Lin J. A pan-cancer-bioinformatic-based literature review of TRPM7 in cancers. Pharmacol Ther. 2022;240: 108302. 36332746 10.1016/j.pharmthera.2022.108302

[58] Liu H, Li Y. Potential roles of cornichon family AMPA receptor auxiliary protein 4 (CNIH4) in head and neck squamous cell carcinoma. Cancer Biomark. 2022;35:439–50. 10.3233/cbm-220143. 36404537 10.3233/CBM-220143 PMC12364253

[59] Liu H, Tang T. Pan-cancer genetic analysis of cuproptosis and copper metabolism-related gene set. Front Oncol. 2022;12: 952290. 36276096 10.3389/fonc.2022.952290 PMC9582932

[60] Liu H, Tang T. Descriptive pan-cancer genetic analysis of disulfidptosis-related gene set. bioRxiv; 2023, 2023.2002. 2025.529997. 10.1016/j.cancergen.2023.10.001 37879141

[61] Liu H, Tang T. A bioinformatic study of IGFBPs in glioma regarding their diagnostic, prognostic, and therapeutic prediction value. Am J Transl Res. 2023;15:2140. 37056850 PMC10086936

[62] Liu H, Tang T. Pan-cancer genetic analysis of disulfidptosis-related gene set. Cancer Genet. 2023;278:91–103. 37879141 10.1016/j.cancergen.2023.10.001

[63] Liu H, Tang T. MAPK signaling pathway-based glioma subtypes, machine-learning risk model, and key hub proteins identification. Sci Rep. 2023;13:19055. 37925483 10.1038/s41598-023-45774-0 PMC10625624

[64] Liu H, Weng J. A comprehensive bioinformatic analysis of cyclin-dependent kinase 2 (CDK2) in glioma. Gene. 2022;822: 146325. 35183683 10.1016/j.gene.2022.146325

[65] Liu H, Weng J. A pan-cancer bioinformatic analysis of RAD51 regarding the values for diagnosis, prognosis, and therapeutic prediction. Front Oncol. 2022;12: 858756. 35359409 10.3389/fonc.2022.858756 PMC8960930

[66] Liu H, Xie R, Dai Q, Fang J, Xu Y, Li B. Exploring the mechanism underlying hyperuricemia using comprehensive research on multi-omics. Sci Rep. 2023;13:7161. 37138053 10.1038/s41598-023-34426-y PMC10156710

[67] Liu H, Dong A, Rasteh AM, Wang P, Weng J. Identification of the novel exhausted T cell CD8 + markers in breast cancer. Sci Rep. 2024;14:19142. 10.1038/s41598-024-70184-1. 39160211 10.1038/s41598-024-70184-1 PMC11333736

[68] Zhou J, Li YS, Chien S. Shear stress-initiated signaling and its regulation of endothelial function. Arterioscler Thromb Vasc Biol. 2014;34:2191–8. 10.1161/atvbaha.114.303422. 24876354 10.1161/ATVBAHA.114.303422 PMC4169328

[69] Albarrán-Juárez J, Iring A, Wang S, Joseph S, Grimm M, Strilic B, Wettschureck N, Althoff TF, Offermanns S. Piezo1 and G(q)/G(11) promote endothelial inflammation depending on flow pattern and integrin activation. J Exp Med. 2018;215:2655–72. 10.1084/jem.20180483. 30194266 10.1084/jem.20180483 PMC6170174

[70] Amadio P, Zarà M, Sandrini L, Ieraci A, Barbieri SS. Depression and cardiovascular disease: the viewpoint of platelets. Int J Mol Sci. 2020. 10.3390/ijms21207560. 33066277 10.3390/ijms21207560 PMC7589256

[71] Izzi B, Tirozzi A, Cerletti C, Donati MB, de Gaetano G, Hoylaerts MF, Iacoviello L, Gialluisi A. Beyond haemostasis and thrombosis: platelets in depression and its co-morbidities. Int J Mol Sci. 2020. 10.3390/ijms21228817. 33233416 10.3390/ijms21228817 PMC7700239

[72] Morel-Kopp MC, McLean L, Chen Q, Tofler GH, Tennant C, Maddison V, Ward CM. The association of depression with platelet activation: evidence for a treatment effect. J Thromb Haemost JTH. 2009;7:573–81. 10.1111/j.1538-7836.2009.03278.x. 19192119 10.1111/j.1538-7836.2009.03278.x

[73] Wang H, He Y, Sun Z, Ren S, Liu M, Wang G, Yang J. Microglia in depression: an overview of microglia in the pathogenesis and treatment of depression. J Neuroinflamm. 2022;19:132. 10.1186/s12974-022-02492-0. 10.1186/s12974-022-02492-0 PMC9168645 35668399

[74] Han X, Xu T, Fang Q, Zhang H, Yue L, Hu G, Sun L. Quercetin hinders microglial activation to alleviate neurotoxicity via the interplay between NLRP3 inflammasome and mitophagy. Redox Biol. 2021;44: 102010. 10.1016/j.redox.2021.102010. 34082381 10.1016/j.redox.2021.102010 PMC8182123

[75] Lai JJ, Cruz FM, Rock KL. Immune sensing of cell death through recognition of histone sequences by C-type lectin-receptor-2d causes inflammation and tissue injury. Immunity. 2020;52:123-135.e126. 10.1016/j.immuni.2019.11.013. 31859049 10.1016/j.immuni.2019.11.013 PMC6962543

[76] Pantazatos SP, Huang YY, Rosoklija GB, Dwork AJ, Arango V, Mann JJ. Whole-transcriptome brain expression and exon-usage profiling in major depression and suicide: evidence for altered glial, endothelial and ATPase activity. Mol Psychiatry. 2017;22:760–73. 10.1038/mp.2016.130. 27528462 10.1038/mp.2016.130 PMC5313378

[77] Ji C, Tang Y, Zhang Y, Li C, Liang H, Ding L, Xia X, Xiong L, Qi XR, Zheng JC. Microglial glutaminase 1 deficiency mitigates neuroinflammation associated depression. Brain Behav Immun. 2022;99:231–45. 10.1016/j.bbi.2021.10.009. 34678461 10.1016/j.bbi.2021.10.009

[78] Li C, Huang J, Cheng YC, Zhang YW. Traditional Chinese Medicine in depression treatment: from molecules to systems. Front Pharmacol. 2020;11:586. 10.3389/fphar.2020.00586. 32457610 10.3389/fphar.2020.00586 PMC7221138

[79] Ostuzzi G, Matcham F, Dauchy S, Barbui C, Hotopf M. Antidepressants for the treatment of depression in people with cancer. Cochrane Database Syst Rev. 2018;4:Cd011006. 10.1002/14651858.CD011006.pub3. 29683474 10.1002/14651858.CD011006.pub3 PMC6494588

[80] Zhang T, Wei W, Chang S, Liu N, Li H. Integrated network pharmacology and comprehensive bioinformatics identifying the mechanisms and molecular targets of yizhiqingxin formula for treatment of comorbidity with Alzheimer’s disease and depression. Front Pharmacol. 2022;13: 853375. 10.3389/fphar.2022.853375. 35548356 10.3389/fphar.2022.853375 PMC9081443

[81] Vengeliene V, Bilbao A, Molander A, Spanagel R. Neuropharmacology of alcohol addiction. Br J Pharmacol. 2008;154:299–315. 10.1038/bjp.2008.30. 18311194 10.1038/bjp.2008.30 PMC2442440

[82] Miguel-Hidalgo JJ, Waltzer R, Whittom AA, Austin MC, Rajkowska G, Stockmeier CA. Glial and glutamatergic markers in depression, alcoholism, and their comorbidity. J Affect Disord. 2010;127:230–40. 10.1016/j.jad.2010.06.003. 20580095 10.1016/j.jad.2010.06.003 PMC2975814

[83] Fluharty M, Taylor AE, Grabski M, Munafò MR. The association of cigarette smoking with depression and anxiety: a systematic review. Nicotine Tobacco Res. 2017;19:3–13. 10.1093/ntr/ntw140. 10.1093/ntr/ntw140 PMC5157710 27199385

[84] Smethells JR, Burroughs D, Saykao A, Pentel PR, Rezvani AH, LeSage MG. The reinforcement threshold and elasticity of demand for nicotine in an adolescent rat model of depression. Drug Alcohol Depend. 2021;219: 108433. 10.1016/j.drugalcdep.2020.108433. 33310485 10.1016/j.drugalcdep.2020.108433 PMC7855441

[85] Hidalgo A, Libby P, Soehnlein O, Aramburu IV, Papayannopoulos V, Silvestre-Roig C. Neutrophil extracellular traps: from physiology to pathology. Cardiovasc Res. 2022;118:2737–53. 10.1093/cvr/cvab329. 34648022 10.1093/cvr/cvab329 PMC9586562

[86] Papayannopoulos V. Neutrophil extracellular traps in immunity and disease. Nat Rev Immunol. 2018;18:134–47. 10.1038/nri.2017.105. 28990587 10.1038/nri.2017.105

[87] Oliveira AI, Pinho C, Sarmento B, Dias AC. Neuroprotective **a**ctivity of *Hypericum**perforatum* and its major components. Front Plant Sci. 2016;7:1004. 10.3389/fpls.2016.01004. 27462333 10.3389/fpls.2016.01004 PMC4939296

[88] Apaydin EA, Maher AR, Shanman R, Booth MS, Miles JN, Sorbero ME, Hempel S. A systematic review of St John’s wort for major depressive disorder. Syst Rev. 2016;5:148. 10.1186/s13643-016-0325-2. 27589952 10.1186/s13643-016-0325-2 PMC5010734

[89] Sell TS, Belkacemi T, Flockerzi V, Beck A. Protonophore properties of hyperforin are essential for its pharmacological activity. Sci Rep. 2014;4:7500. 10.1038/srep07500. 25511254 10.1038/srep07500 PMC4266863

[90] Caldeira GI, Gouveia LP, Serrano R, Silva OD. *Hypericum* genus as a natural source for biologically active compounds. Plants (Basel, Switzerland). 2022. 10.3390/plants11192509. 36235373 10.3390/plants11192509 PMC9573133

[91] Blume J, Douglas SD, Evans DL. Immune suppression and immune activation in depression. Brain Behav Immun. 2011;25:221–9. 10.1016/j.bbi.2010.10.008. 20955778 10.1016/j.bbi.2010.10.008 PMC3025086

[92] Berk M, Williams LJ, Jacka FN, O’Neil A, Pasco JA, Moylan S, Allen NB, Stuart AL, Hayley AC, Byrne ML, et al. So depression is an inflammatory disease, but where does the inflammation come from? BMC Med. 2013;11:200. 10.1186/1741-7015-11-200. 24228900 10.1186/1741-7015-11-200 PMC3846682

[93] Beurel E, Medina-Rodriguez EM, Jope RS. Targeting the adaptive immune system in depression: focus on T helper 17 cells. Pharmacol Rev. 2022;74:373–86. 10.1124/pharmrev.120.000256. 35302045 10.1124/pharmrev.120.000256 PMC8973514

[94] Simon MS, Schiweck C, Arteaga-Henríquez G, Poletti S, Haarman BCM, Dik WA, Schwarz M, Vrieze E, Mikova O, Joergens S, et al. Monocyte mitochondrial dysfunction, inflammaging, and inflammatory pyroptosis in major depression. Prog Neuropsychopharmacol Biol Psychiatry. 2021;111: 110391. 10.1016/j.pnpbp.2021.110391. 34171401 10.1016/j.pnpbp.2021.110391

[95] Chen S, Tang Y, Gao Y, Nie K, Wang H, Su H, Wang Z, Lu F, Huang W, Dong H. Antidepressant potential of quercetin and its glycoside derivatives: a comprehensive review and update. Front Pharmacol. 2022;13: 865376. 10.3389/fphar.2022.865376. 35462940 10.3389/fphar.2022.865376 PMC9024056

[96] Silva Dos Santos J, Gonçalves Cirino JP, de Oliveira Carvalho P, Ortega MM. The pharmacological action of kaempferol in central nervous system diseases: a review. Front Pharmacol. 2020;11: 565700. 10.3389/fphar.2020.565700. 33519431 10.3389/fphar.2020.565700 PMC7838523

[97] Gao W, Wang W, Peng Y, Deng Z. Antidepressive effects of kaempferol mediated by reduction of oxidative stress, proinflammatory cytokines and up-regulation of AKT/β-catenin cascade. Metab Brain Dis. 2019;34:485–94. 10.1007/s11011-019-0389-5. 30762138 10.1007/s11011-019-0389-5

[98] Achour M, Ferdousi F, Sasaki K, Isoda H. Luteolin modulates neural stem cells fate determination: in vitro study on human neural stem cells, and in vivo study on LPS-induced depression mice model. Front Cell Dev Biol. 2021;9: 753279. 10.3389/fcell.2021.753279. 34790666 10.3389/fcell.2021.753279 PMC8591246

[99] Sur B, Lee B. Luteolin reduces fear, anxiety, and depression in rats with post-traumatic stress disorder. Anim Cells Syst. 2022;26:174–82. 10.1080/19768354.2022.2104925. 10.1080/19768354.2022.2104925 PMC9423864 36046028

[100] Thiam HR, Wong SL, Wagner DD, Waterman CM. Cellular mechanisms of NETosis. Annu Rev Cell Dev Biol. 2020;36:191–218. 10.1146/annurev-cellbio-020520-111016. 32663035 10.1146/annurev-cellbio-020520-111016 PMC8499668

[101] Castanheira FVS, Kubes P. Neutrophils and NETs in modulating acute and chronic inflammation. Blood. 2019;133:2178–85. 10.1182/blood-2018-11-844530. 30898862 10.1182/blood-2018-11-844530

[102] Glaviano A, Foo ASC, Lam HY, Yap KCH, Jacot W, Jones RH, Eng H, Nair MG, Makvandi P, Geoerger B, et al. PI3K/AKT/mTOR signaling transduction pathway and targeted therapies in cancer. Mol Cancer. 2023;22:138. 10.1186/s12943-023-01827-6. 37596643 10.1186/s12943-023-01827-6 PMC10436543

[103] Ji Y, Luo J, Zeng J, Fang Y, Liu R, Luan F, Zeng N. Xiaoyao pills ameliorate depression-like behaviors and oxidative stress induced by olfactory bulbectomy in rats via the activation of the PIK3CA-AKT1-NFE2L2/BDNF signaling pathway. Front Pharmacol. 2021;12: 643456. 10.3389/fphar.2021.643456. 33935736 10.3389/fphar.2021.643456 PMC8082504

[104] Islam F, Roy S, Zehravi M, Paul S, Sutradhar H, Yaidikar L, Kumar BR, Dogiparthi LK, Prema S, Nainu F, et al. Polyphenols targeting MAP kinase signaling pathway in neurological diseases: understanding molecular mechanisms and therapeutic targets. Mol Neurobiol. 2024;61:2686–706. 10.1007/s12035-023-03706-z. 37922063 10.1007/s12035-023-03706-z

[105] Bretones G, Delgado MD, León J. Myc and cell cycle control. Biochem Biophys Acta. 2015;1849:506–16. 10.1016/j.bbagrm.2014.03.013. 24704206 10.1016/j.bbagrm.2014.03.013

[106] Zaytseva O, Kim NH, Quinn LM. MYC in Brain Development and Cancer. Int J Mol Sci. 2020. 10.3390/ijms21207742. 33092025 10.3390/ijms21207742 PMC7588885

[107] Marinkovic T, Marinkovic D. Obscure involvement of MYC in neurodegenerative diseases and neuronal repair. Mol Neurobiol. 2021;58:4169–77. 10.1007/s12035-021-02406-w. 33954904 10.1007/s12035-021-02406-w

[108] Wells A. EGF receptor. Int J Biochem Cell Biol. 1999;31:637–43. 10.1016/s1357-2725(99)00015-1. 10404636 10.1016/s1357-2725(99)00015-1

[109] Endo M, Cerione RA. The brain-specific splice variant of the CDC42 GTPase works together with the kinase ACK to downregulate the EGF receptor in promoting neurogenesis. J Biol Chem. 2022;298: 102564. 10.1016/j.jbc.2022.102564. 36206843 10.1016/j.jbc.2022.102564 PMC9663532

[110] Jin K, Sun Y, Xie L, Batteur S, Mao XO, Smelick C, Logvinova A, Greenberg DA. Neurogenesis and aging: FGF-2 and HB-EGF restore neurogenesis in hippocampus and subventricular zone of aged mice. Aging Cell. 2003;2:175–83. 10.1046/j.1474-9728.2003.00046.x. 12882410 10.1046/j.1474-9728.2003.00046.x

[111] Zuehlke AD, Beebe K, Neckers L, Prince T. Regulation and function of the human HSP90AA1 gene. Gene. 2015;570:8–16. 10.1016/j.gene.2015.06.018. 26071189 10.1016/j.gene.2015.06.018 PMC4519370

[112] Brites D, Fernandes A. Neuroinflammation and depression: microglia activation, extracellular microvesicles and microRNA dysregulation. Front Cell Neurosci. 2015;9:476. 10.3389/fncel.2015.00476. 26733805 10.3389/fncel.2015.00476 PMC4681811

[113] Wu Z, Cai Z, Shi H, Huang X, Cai M, Yuan K, Huang P, Shi G, Yan T, Li Z. Effective biomarkers and therapeutic targets of nerve-immunity interaction in the treatment of depression: an integrated investigation of the miRNA-mRNA regulatory networks. Aging. 2022;14:3569–96. 10.18632/aging.204030. 35468096 10.18632/aging.204030 PMC9085226

[114] Lopez JP, Fiori LM, Cruceanu C, Lin R, Labonte B, Cates HM, Heller EA, Vialou V, Ku SM, Gerald C, et al. MicroRNAs 146a/b-5 and 425–3p and 24–3p are markers of antidepressant response and regulate MAPK/Wnt-system genes. Nat Commun. 2017;8:15497. 10.1038/ncomms15497. 28530238 10.1038/ncomms15497 PMC5477510

[115] Hodan R, Charville GW, Ladabaum U. Hereditary inflammatory fibroid polyps caused by germline pathogenic variants in PDGFRA: refining PDGFRA-mutation syndrome. Cancer Genet. 2021;256:106–9. 34107389 10.1016/j.cancergen.2021.05.003

[116] Hutchison J, Cohen Z, Onyeagucha BC, Funk J, Nelson MA. How microRNAs influence both hereditary and inflammatory-mediated colon cancers. Cancer Genet. 2013;206:309–16. 24042167 10.1016/j.cancergen.2013.06.005 PMC3893936

[117] Li R, Mukherjee MB, Jin Z, Liu H, Lin K, Liu Q, Dilger JP, Lin J. The potential effect of general anesthetics in cancer surgery: meta-analysis of postoperative metastasis and inflammatory cytokines. Cancers. 2023;15:2759. 37345096 10.3390/cancers15102759 PMC10216624

[118] Owusu-Brackett N, Johnson R, Schindel DT, Koduru P, Cope-Yokoyama S. A novel ALK rearrangement in an inflammatory myofibroblastic tumor in a neonate. Cancer Genet. 2013;206:353–6. 24290361 10.1016/j.cancergen.2013.10.002

[119] García-Montero C, Ortega MA, Alvarez-Mon MA, Fraile-Martinez O, Romero-Bazán A, Lahera G, Montes-Rodríguez JM, Molina-Ruiz RM, Mora F, Rodriguez-Jimenez R, et al. The problem of malnutrition associated with major depressive disorder from a sex-gender perspective. Nutrients. 2022. 10.3390/nu14051107. 35268082 10.3390/nu14051107 PMC8912662

[120] Slavich GM, Sacher J. Stress, sex hormones, inflammation, and major depressive disorder: extending social signal transduction theory of depression to account for sex differences in mood disorders. Psychopharmacology. 2019;236:3063–79. 10.1007/s00213-019-05326-9. 31359117 10.1007/s00213-019-05326-9 PMC6821593

[121] Kuehner C. Why is depression more common among women than among men? Lancet Psychiatry. 2017;4:146–58. 10.1016/s2215-0366(16)30263-2. 27856392 10.1016/S2215-0366(16)30263-2

[122] Ghazanfarpour M, Sadeghi R, Latifnejad Roudsari R, Khadivzadeh T, Khorsand I, Afiat M, Esmaeilizadeh M. Effects of flaxseed and *Hypericum**perforatum* on hot flash, vaginal atrophy and estrogen-dependent cancers in menopausal women: a systematic review and meta-analysis. Avicenna J Phytomed. 2016;6:273–83. 27462550 PMC4930534

[123] Hastie T, Tibshirani R, Friedman J. The elements of statistical learning: data mining, inference, and prediction; 2017.

